# HIV testing and treatment coverage achieved after 4 years across 14 urban and peri-urban communities in Zambia and South Africa: An analysis of findings from the HPTN 071 (PopART) trial

**DOI:** 10.1371/journal.pmed.1003067

**Published:** 2020-04-02

**Authors:** Sian Floyd, Kwame Shanaube, Blia Yang, Ab Schaap, Sam Griffith, Mwelwa Phiri, David Macleod, Rosa Sloot, Kalpana Sabapathy, Virginia Bond, Peter Bock, Helen Ayles, Sarah Fidler, Richard Hayes, the HPTN 071 (PopART) study team

**Affiliations:** 1 Department of Infectious Disease Epidemiology, London School of Hygiene & Tropical Medicine, London, United Kingdom; 2 Zambart, University of Zambia School of Medicine, Lusaka, Zambia; 3 Desmond Tutu TB Centre, Department of Paediatrics and Child Health, Stellenbosch University, Stellenbosch, South Africa; 4 FHI 360, HIV Prevention Trials Network, Durham, North Carolina, United States of America; 5 Department of Global Health and Policy, London School of Hygiene & Tropical Medicine, London, United Kingdom; 6 Department of Clinical Research, London School of Hygiene & Tropical Medicine, London, United Kingdom; 7 HIV Clinical Trials Unit, Imperial College London, London, United Kingdom; University of California, San Francisco, UNITED STATES

## Abstract

**Background:**

In 2014, the Joint United Nations Programme on HIV/AIDS (UNAIDS) set the 90-90-90 targets: that 90% of people living with HIV know their HIV status, that 90% of those who know their HIV-positive status are on antiretroviral therapy (ART), and that 90% of those on treatment are virally suppressed. The aim was to reach these targets by 2020. We assessed the feasibility of achieving the first two targets, and the corresponding 81% ART coverage target, as part of the HIV Prevention Trials Network (HPTN) 071 Population Effects of Antiretroviral Therapy to Reduce HIV Transmission (PopART) community-randomized trial.

**Methods and findings:**

The study population was individuals aged ≥15 years living in 14 urban and peri-urban “PopART intervention” communities in Zambia and South Africa (SA), with a total population of approximately 600,000 and approximately 15% adult HIV prevalence. Community HIV care providers (CHiPs) delivered the PopART intervention during 2014–2017. This was a combination HIV prevention package including universal home-based HIV testing, referral of HIV-positive individuals to government HIV clinic services that offered universal ART (Arm A) or ART according to national guidelines (Arm B), and revisits to HIV-positive individuals to support linkage to HIV care and retention on ART. The intervention was delivered in 3 “rounds,” each about 15 months long, during which CHiPs visited all households and aimed to contact all individuals aged ≥15 years at least once.

In Arm A in Round 3 (R3), 67% (41,332/61,402) of men and 86% (56,345/65,896) of women in Zambia and 56% (17,813/32,095) of men and 71% (24,461/34,514) of women in SA participated in the intervention, among 193,907 residents aged ≥15 years. Following participation, HIV status was known by 90% of men and women in Zambia and by 78% of men and 85% of women in SA. The median time from CHiP referral of HIV-positive individuals to ART initiation was approximately 3 months. By the end of R3, an estimated 95% of HIV-positive women and 85% of HIV-positive men knew their HIV status, and among these individuals, approximately 90% of women and approximately 85% of men were on ART. ART coverage among all HIV-positive individuals was approximately 85% in women and approximately 75% in men, up from about 45% at the start of the study. ART coverage was lowest among men aged 18 to 34 and women aged 15 to 24 years, and among mobile individuals/in-migrants. Findings from Arm B were similar. The main limitations to our study were that estimates of testing and treatment coverage among men relied on considerable extrapolation because, in each round, approximately one-third of men did not participate in the PopART intervention; that our findings are for a service delivery model that was relatively intensive; and that we did not have comparable data from the 7 “standard-of-care” (Arm C) communities.

**Conclusions:**

Our study showed that very high HIV testing and treatment coverage can be achieved through persistent delivery of universal testing, facilitated linkage to HIV care, and universal treatment services. The ART coverage target of 81% was achieved overall, after 4 years of delivery of the PopART intervention, though important gaps remained among men and young people. Our findings are consistent with previously reported findings from southern and east Africa, extending their generalisability to urban settings with high rates of in-migration and mobility and to Zambia and SA.

**Trial registration:**

ClinicalTrials.gov NCT01900977.

## Abstract 2

Sian Floyd and co-authors study progress towards goals for population HIV testing and treatment in South Africa and Zambia.

## Author summary

**Why was this study done?:**

- The HIV Prevention Trials Network (HPTN) 071 Population Effects of Antiretroviral Therapy to Reduce HIV Transmission (PopART) trial aimed to establish whether universal testing and universal treatment for HIV could reduce population-level HIV incidence in a generalised HIV epidemic.
- In 2014, the Joint United Nations Programme on HIV/AIDS (UNAIDS) set targets that, by 2020, 90% of people living with HIV know their HIV status, that 90% of those who know their HIV-positive status are on antiretroviral therapy (ART), and that 90% of those on treatment are virally suppressed (the 90-90-90 targets).
- We assessed the feasibility of achieving these targets as part of the HPTN 071 (PopART) community-randomised trial.
- The study population was individuals aged ≥15 years living in 14 urban and peri-urban “PopART” intervention communities in Zambia and the Western Cape of South Africa (SA), with a total population of approximately 600,000 and HIV prevalence of around 15%.

**What did the researchers do and find?:**

- Community HIV care providers (CHiPs) delivered the PopART intervention during 2014–2017 inclusive. This was a combination HIV prevention package including universal home-based HIV testing, referral of HIV-positive individuals to government HIV clinic services that offered universal ART (Arm A, 7 communities) or ART according to national guidelines (Arm B, 7 communities), and revisits to HIV-positive individuals to support linkage to HIV care and retention on ART.
- High levels of HIV testing and treatment coverage were achieved in communities that received the PopART intervention, especially among women. After 4 years, approximately 95% of HIV-positive women and approximately 85% of HIV-positive men knew their HIV status, up from about 55% at the start of the intervention. Among individuals who knew their HIV-positive status after 4 years, approximately 90% of women and approximately 85% of men were on ART, up from about 80% at the start of the intervention. In the total HIV-positive population—both men and women—the cumulative 90–90 target of 81% ART coverage was met, up from around 45% at the start of the study. Viral suppression among individuals who were taking ART was high, at around 90% in Zambian study communities and around 80%–90% in SA study communities.
- Testing and treatment coverage increased in each intervention round, but progress was slowed by high rates of mobility and in-migration among both men and women. At the start of a round of intervention, mobile individuals and in-migrants had lower knowledge of HIV-positive status and ART coverage than “stable” residents. Progress was also slowed by the relative difficulty of contacting men at home, which persisted throughout the study.
- The main remaining gap in coverage against the first and second of the 90-90-90 targets after 4 years of intervention was among men aged 18–34 years and women aged 15–24 years. This reflected fewer prior opportunities for facility-based HIV testing, relatively high HIV incidence compared with HIV prevalence, and relatively high mobility and in-migration rates. Participation in the PopART intervention and uptake of HIV testing were similar to or higher among younger individuals compared with those aged 35–54 years.

**What do these findings mean?:**

- Very high HIV testing and treatment coverage can be achieved through persistent delivery of universal testing, facilitated linkage to HIV care, and universal treatment services.
- Our findings are consistent with previously reported findings from southern and east Africa, extending their generalisability to urban settings with high rates of in-migration and mobility and to Zambia and SA.

## Introduction

In 2014, the Joint United Nations Programme on HIV/AIDS (UNAIDS) set the so-called “90-90-90” global targets: that 90% of HIV-positive individuals know their HIV-positive status, 90% of those who know their HIV-positive status are on antiretroviral therapy (ART), and 90% of those taking ART are virally suppressed [1]. The targets were set for 2020, with the aim of reducing the number of new HIV infections among adults from approximately 2 million in 2015 to approximately 500,000 per year in 2020. The 90-90-90 targets correspond cumulatively to 81% of HIV-positive individuals being on ART and 73% being virally suppressed.

By 2017–2018, data from nationally representative Population-based HIV Impact Assessment (PHIA) surveys across 10 countries in sub-Saharan Africa showed substantial progress toward the cumulative 90-90-90 target in 7 countries [2,3]. The cumulative target had been met in Namibia, was close to being met in eSwatini and Malawi, and was approximately 10% below target in Zambia, Zimbabwe, Lesotho, and Uganda. Progress fell short of the target in Tanzania, Cameroon, and Ivory Coast. Across 9 of the 10 countries, both the percentage on ART among individuals who knew their HIV-positive status (“second 90”) and the percentage with viral suppression among those taking ART (“third 90”) was on or close to target, overall and for both men and women [2]. The key gap that remained to be closed was for the percentage of HIV-positive individuals who knew their HIV-positive status (“first 90”), with the gap being wider for men than for women and wider for younger than older individuals.

Four cluster-randomised trials (CRTs) of the effect of combination HIV prevention packages, which included universal HIV testing and universal treatment alongside pre-existing service provision, on HIV incidence have also provided evidence about coverage against the 90-90-90 targets. These are the ANRS 12249 Treatment as Prevention (TasP) trial in South Africa (SA), the Sustainable East Africa Research in Community Health (SEARCH) trial in Uganda and Kenya, the Botswana Combination Prevention Project (BCPP)/YaTsie trial in Botswana, and the HIV Prevention Trials Network (HPTN) 071 Population Effects of Antiretroviral Therapy to Reduce HIV Transmission (PopART) trial in Zambia and the Western Cape of SA. At study end, after 3 years of intervention, the first and second 90 targets and the cumulative 90-90-90 target of 73% were achieved in the SEARCH trial, and in the BCPP/YaTsie trial 95-95-95 was reached [4,5]. In contrast, in the ANRS 12249 TasP trial coverage against the second 90 target remained relatively low at study end, after 2 years of intervention [6].

Previously, we reported the progress of the PopART universal testing and treatment (UTT) intervention toward the 90-90-90 targets after the first 2 years of the study (2014–2016). At that time, the first 90 target was close to being met, and an estimated approximately 80% of HIV-positive individuals who knew their HIV-positive status were on ART [7]. Challenges to reaching the first and second 90 targets included the following: difficulties in contacting men; high rates of mobility and in-migration to study communities that meant that, in each year, a considerable proportion of the population was newly resident and had lower testing-and-treatment coverage than previously resident individuals; that approximately 20% of individuals who participated in the wider intervention declined HIV testing; and slower-than-targeted linkage to HIV care [7,8].

In this paper, we report on progress toward reaching the first and second 90 targets after 4 years of delivering the PopART UTT intervention (2014–2017) and the extent to which it was possible to close the gaps in coverage that were identified during 2014–2016 with more time, accumulated experience, and new approaches. We also compare testing and treatment coverage between the 2 intervention trial arms of the PopART study to provide insight into the primary finding of the trial that HIV incidence in the PopART UTT intervention trial arm was around 10% lower than in the standard-of-care trial arm, but it was around 30% lower in the “intermediate” PopART intervention trial arm [9].

## Methods

### Setting and trial design

**Fig 1:**
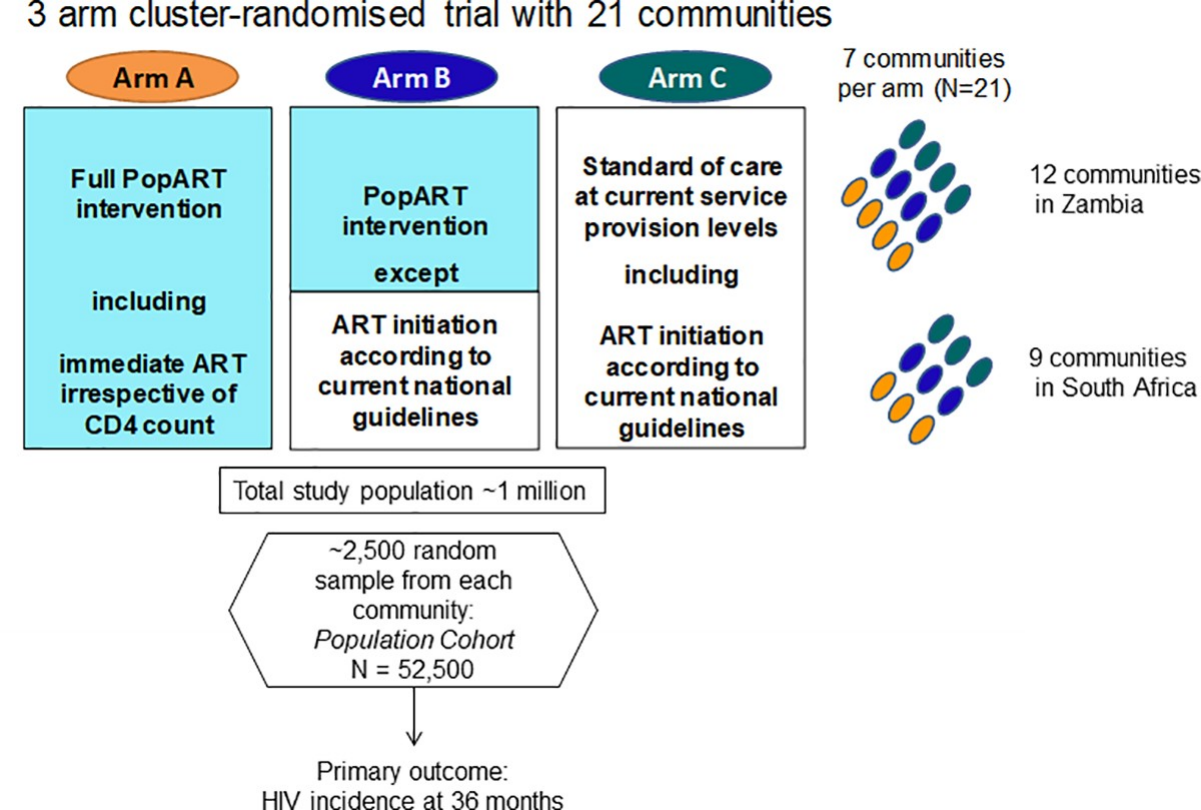
Summary of the HPTN 071 (PopART) trial design. CD4, cluster of differentiation 4; ART, antiretroviral therapy.

The study population consisted of individuals aged ≥15 years living in the 14 intervention communities of the HPTN 071 (PopART) CRT, 8 in Zambia and 6 in the Western Cape of SA. The communities were urban or peri-urban, with a total population across the 14 communities of approximately 600,000 [10]. HIV prevalence was around 10% in men and around 20% in women, with peak HIV prevalence among men aged 40 to 49 years and women aged 35 to 44 years [7]. Seven communities were randomised to receive the “full” PopART intervention, including UTT (Arm A, described below), and 7 communities were randomised to receive the “intermediate” PopART intervention, with universal testing but with ART offered according to national guidelines (Arm B). Seven communities were randomised to receive standard of care (the control, Arm C), and the trial design is summarised in Fig 1. The primary outcome of the study (HIV incidence) and secondary outcomes were measured in a population cohort study, with sample size chosen to have good study power to make comparisons between both of Arms A and B with Arm C [9,10].

### PopART intervention

Community HIV care providers (CHiPs) delivered the PopART intervention during 2014–2017 inclusive. This consisted of offering universal home-based HIV testing, referral of HIV-positive individuals to government HIV clinic services that offered universal ART (Arm A) or ART according to national guidelines (Arm B), and revisits to HIV-positive individuals to support linkage to HIV care and retention on ART, as well as various other services that together made up a combination HIV prevention package, as described previously [7,11]. Arm B communities transitioned to offering universal treatment in mid-2016, so service delivery was the same as in Arm A for the last 18 months of the intervention.

Before the start of the PopART intervention, each intervention community was divided into “zones” containing approximately 500 households. CHiPs worked in teams of 2, with each team responsible for one zone. They delivered the intervention in “rounds,” during which they visited all households, offered to explain the intervention, and asked permission to enumerate (list) all household members. The first round (Round 1 [R1]) was November 2013 to June 2015, the second (Round 2 [R2]) was June 2015 to October 2016, and the third (Round 3 [R3]) was October 2016 to December 2017. Within each round, CHiPs aimed to contact all individuals aged ≥15 years (R2–R3) or ≥18 years (R1) at least once to offer them intervention services. No “incentives” to participation were provided.

During R2–R3, new strategies were implemented to try to increase participation, HIV testing uptake, and linkage to HIV care, contributing to improvements across this cascade [7]. In some zones, CHiPs periodically supplemented their household visits with “zonal campaigns” during which they offered services in a non-household setting, such as at markets and transport hubs [12]; in most zones, they worked shifts early and late in the day and on weekends [13]. In Zambia in February 2017, the novel option of HIV self-testing was introduced to half of the CHiP zones in 2 Arm A and 2 Arm B communities, in a nested randomised trial. Following evidence that this increased testing uptake among men [14], the option to self-test was rolled out to all CHiP zones in Zambia during July–December 2017. For individuals who had been referred but not yet linked to care, or who had dropped out of care, household follow-up visits and/or phone calls were made to provide additional counselling and to offer to accompany an individual to the clinic. Coordination between the CHiPs and the clinic was improved, and various clinic improvements were made.

### Data collection, outcomes, and explanatory variables

CHiPs recorded information on electronic registers, as described elsewhere [7,11]. For each round, individuals were considered to have participated in the intervention if they verbally consented to participate and health counselling was done. They were “known HIV positive” if during that round they either self-reported that they were HIV positive or accepted the offer of HIV testing and tested HIV positive. They were considered to “know their HIV status” if they either self-reported HIV positive, tested for HIV with CHiPs, or self-reported that they had tested HIV negative in the last 3 months. For all individuals who were “known HIV positive,” CHiPs were expected to collect updated information at follow-up visits about whether an individual was still resident in the zone, and if the individual was contacted, they collected self-reported information on ART initiation and retention.

The main outcomes were participation in the intervention, knowledge of HIV status among those who participated, the percentage of participants who were known by the CHiPs to be HIV positive, estimates of coverage against the first and second 90 targets among participants and with extrapolation to the total population, and estimates of the time from CHiP referral to ART initiation. These outcomes were prespecified in the HPTN 071 (PopART) statistical analysis plan [15]; they were summarised throughout the study to monitor intervention delivery and were regularly reviewed by the study’s Data Safety and Monitoring Board (DSMB). Explanatory variables were trial arm, country, round of intervention, sex, age group, and residency/participation in previous rounds of intervention.

### Estimation of the number of HIV-positive individuals in the population and coverage against the first and second 90 targets: “Central” estimates and sensitivity analyses

Methods for estimating the number of HIV-positive individuals in the total population (both participants and nonparticipants) and associated coverage against the first and second 90 targets have been described in detail previously [7,16]. They were also set out in the HPTN 071 statistical analysis plan [15] and are summarized in Box 1.

**Box 1. Summary of methods used to estimate coverage against the first two of the UNAIDS 90-90-90 targets**

Among individuals who participated in the intervention in a particular round, we estimated the number of HIV-positive individuals as the number who were “known HIV positive” plus an estimated number among those whose HIV status was not known to CHiPs, assuming that HIV prevalence in the latter group was the same as among those who tested for HIV. We then calculated:

1. the proportion of HIV-positive individuals who knew their HIV-positive status immediately before the household visit of that round as the total who self-reported they were HIV-positive, divided by the estimated number of HIV-positive individuals;
2. the proportion who knew their HIV-positive status immediately after the household visit of that round as the total who were known by the CHiPs to be HIV positive following the round (self-reported HIV positive or tested HIV positive with CHiPs), divided by the estimated number of HIV-positive individuals;
3. the proportion who were on ART immediately after the household visit of that round, among those who knew their HIV-positive status, as the total who self-reported that they were on ART divided by the number who were known by the CHiPs to be HIV positive following the round; and
4. the proportion who were on ART by the end of the round, among those who knew their HIV-positive status, as the total who self-reported that they were on ART at the last CHiP visit made during the round, divided by the number who were known by the CHiPs to be HIV positive and who remained resident in the same zone of the community according to the last information collected during the round.

For “central” estimates of coverage, we extrapolated to the total population (including individuals who did not participate in the intervention in a particular round) by assuming that HIV prevalence was the same in nonparticipants and participants and that knowledge of HIV-positive status and ART uptake among nonparticipants of a round was the same as among participants immediately before they were visited by CHiPs in that round. In sensitivity analysis, we varied 6 key values around the “central” assumptions: HIV prevalence, knowledge of HIV-positive status, and ART uptake, separately for (i) participants whose HIV status was unknown to CHiPs and (ii) nonparticipants. In total, we considered 486 scenarios (2 × 3 × 3 × 3 × 3 × 3, in order of the 6 values above).

All estimates were calculated within strata defined by all combinations of trial arm, round, community, sex, and age group; for Zambia, estimates for R2–R3 were also stratified on participation, residency, and HIV status in previous rounds.

### Estimation of the time from CHiP referral to ART initiation

For each round, we used the Kaplan-Meier “time-to-event” method to estimate the time from first CHiP referral in that round to self-reported ART initiation, among individuals who self-reported they were not on ART on the date of referral. We used data on follow-up visits up to the end of the subsequent round of intervention, giving the potential for all referred individuals to be followed up for at least 12 months after referral. For individuals who were not known to have started ART, we censored their follow-up on the date of the last visit at which they were contacted by CHiPs in person.

### Focus of results presented

All analyses were done separately for each trial arm, country, and round of intervention, with a focus on findings from R3 and on placing those findings in the context of findings from R1–R2. For SA, findings from R1–R2 are presented only for estimates of the time from CHiP referral to ART initiation, due to data collection challenges in R1–R2 [7]. We focus primarily on findings from the 7 Arm A communities that received the PopART UTT intervention during 2014–2017. We follow this with a summary and synthesis of whether the findings were similar or different in Arm B communities, in which universal treatment was offered from mid-2016 following a change to national guidelines on ART eligibility.

### Estimates of the “third 90” from the HPTN 071 (PopART) population cohort study

From November 2013 to March 2015, a randomly selected cohort of adults aged 18 to 44 years was enrolled into the HPTN 071 (PopART) population cohort study, to measure the primary outcome of HIV incidence as well as secondary outcomes including viral suppression among HIV-positive individuals [9]. This was a research study, with written informed consent, with annual follow-up for 3 years and a request for collection of a venous blood sample at each follow-up visit. At the 24-month follow-up, conducted from approximately mid-2016 to approximately mid-2017, viral load was measured in all HIV-positive individuals, with viral load testing conducted at the HPTN Laboratory Centre. In the absence of data on viral suppression from the intervention service delivery data, we used viral load data from the 24-month follow-up of the population cohort study to provide estimates of the “third 90,” i.e., the percentage of HIV-positive individuals with viral suppression, among those on ART. The denominator for our analysis was individuals who tested HIV-positive on laboratory HIV testing of a venous blood sample and had viral load testing done and—as part of the 24-month follow-up interview—self-reported that they had taken ART during the previous month.

### Ethical considerations

The study was approved by the ethics committees of the London School of Hygiene & Tropical Medicine, the University of Zambia, and Stellenbosch University. Individuals gave informed verbal consent to participation in the intervention, and informed written or witnessed consent for HIV testing.

## Results

### Arm A, R3: Households visited and enumerated, total individuals enumerated, and population structure

CHiPs visited 75,472 households during R3, approximately 100% of the total. Consent to enumeration of household members was given by 94% of households, and 193,907 individuals aged ≥15 years were recorded. Among individuals aged ≥15 years, approximately 50% were aged <30 years, and in Zambian communities about one-third were newly resident in the area of the community in which they were living in R3—consistent with R2 findings of high levels of mobility within the communities and in-migration from outside them (Table 1).

**Table 1:** Arm A: Population structure among individuals enumerated as a household member in R3.

	Zambian communities				SA communities			
Individual characteristic	Men		Women		Men		Women	
	(*N* = 61,402)		(*N* = 65,896)		(*N* = 32,095)		(*N* = 34,514)	
Age group (years)	*n*	%	*n*	%	*n*	%	*n*	%
15–17	5,728	9.3	6,608	10.0	1,910	5.9	2,273	6.6
18–19	4,567	7.4	5,455	8.3	1,646	5.1	1,902	5.5
20–24	11,386	18.5	14,463	21.9	5,309	16.5	6,079	17.6
25–29	9,677	15.8	10,541	16.0	5,425	16.9	5,902	17.1
30–34	8,223	13.4	8,059	12.2	5,133	16.0	5,116	14.8
35–39	6,819	11.1	6,149	9.3	3,821	11.9	3,736	10.8
40–44	5,280	8.6	4,404	6.7	3,032	9.4	3,059	8.9
45–49	3,336	5.4	2,797	4.2	2,063	6.4	2,243	6.5
50–54	2,057	3.3	2,413	3.7	1,664	5.2	1,783	5.2
55–59	1,419	2.3	1,678	2.5	985	3.1	1,050	3.0
60–64	1,010	1.6	1,306	2.0	594	1.8	614	1.8
≥65	1,900	3.1	2,023	3.1	513	1.6	757	2.2
**Prior residency and participation (using information from R1 and R2)**								
Previously participated, known HIV positive	2,526	4.1	5,696	8.6	/^1^		/	
Previously participated, tested HIV negative	20,669	33.7	26,027	39.5	/		/	
Previously participated, declined offer of HIV testing	6,482	10.6	7,035	10.7	/		/	
Previously resident, but did not previously participate	8,771	14.3	3,739	5.7	/		/	
Newly resident in area of community in R3 and/or <15 years during R2	22,954	37.4	23,399	35.5	/		/	

^1^The”/” symbol indicates information not available for SA study communities.

Abbreviations: R1, Round 1; R2, Round 2; R3, Round 3; SA, South Africa

### Arm A: Participation in intervention

**Fig 2:**
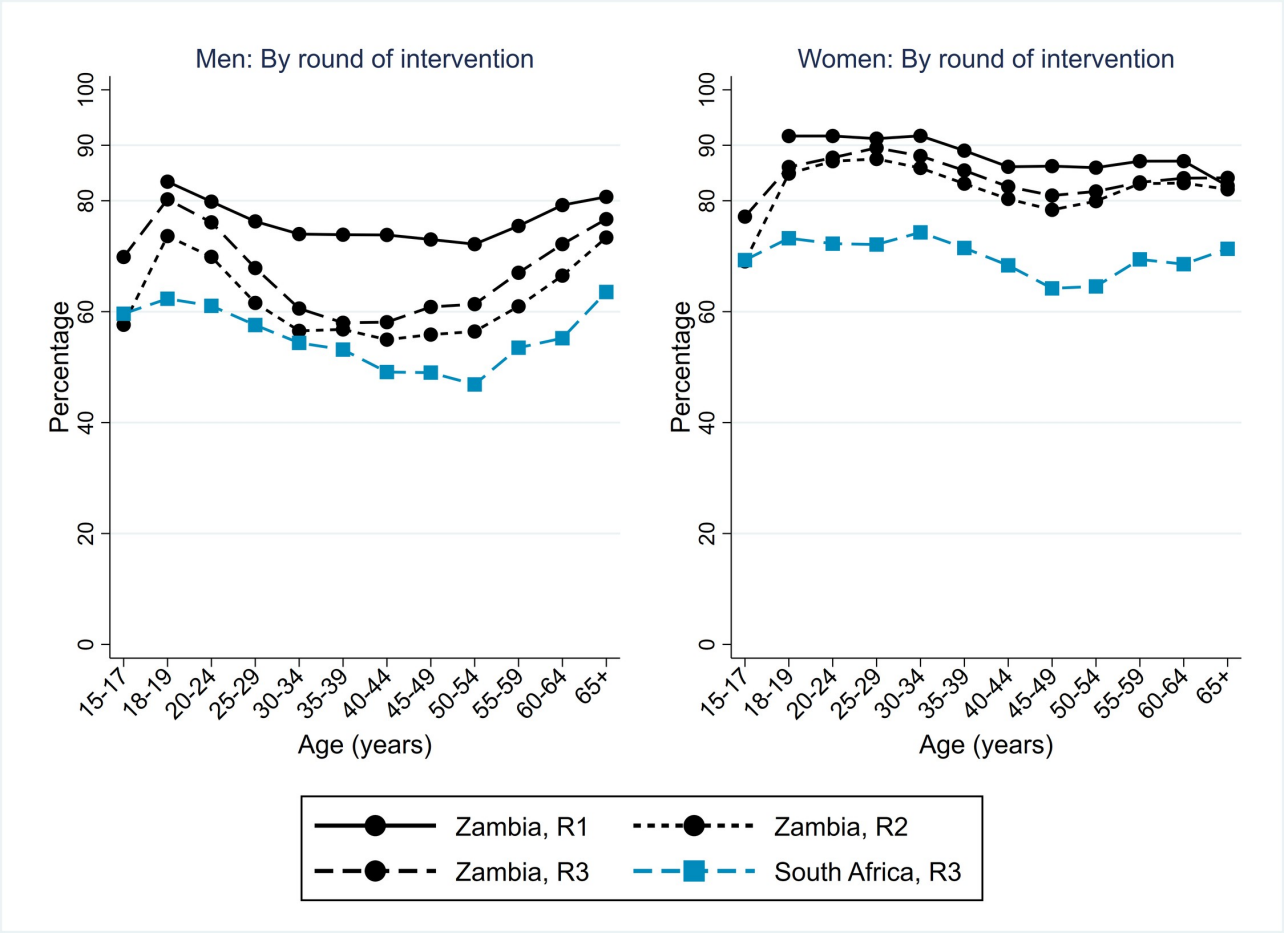
Arm A: Participation in the PopART intervention, Zambian communities R1–R3, and SA communities R3, by sex and age group. R1, Round 1; R2, Round 2; R3, Round 3; SA, South Africa.

During R3, in both Zambian and SA communities it remained challenging for CHiPs to contact men at home. However, in Zambian communities the percentage of men who participated in the intervention in R3 was higher than in R2 across all age groups (Fig 2), with an overall figure of 67% (41,332/61,402) in R3 compared with 62% in R2. Participation was highest among men who had participated in R1 and/or R2 and much higher than among men who were previously resident but did not participate in R1 or R2 (S1 Fig). Among all men who were resident in R3, 78% participated in one of R1–R3. The percentage of men who participated in R3 in SA communities was lower than in Zambian communities, at 56% (17,813/32,095) overall. In both countries, the participation rate was highest among young men (18–24 years) and those ≥60 years.

For women in Zambian communities in R3, participation was very high across the age range and was 86% (56,345/65,896) overall, consistent with findings in R1 and R2 (Fig 2). Among all women who were resident in R3, 92% participated in one of R1–R3. The percentage of women who participated in R3 in SA communities was lower than in Zambian communities, at 71% (24,461/34,514) overall, with relatively little variation by age group.

### Arm A: Knowledge of HIV status among individuals who participated in intervention

**Fig 3:**
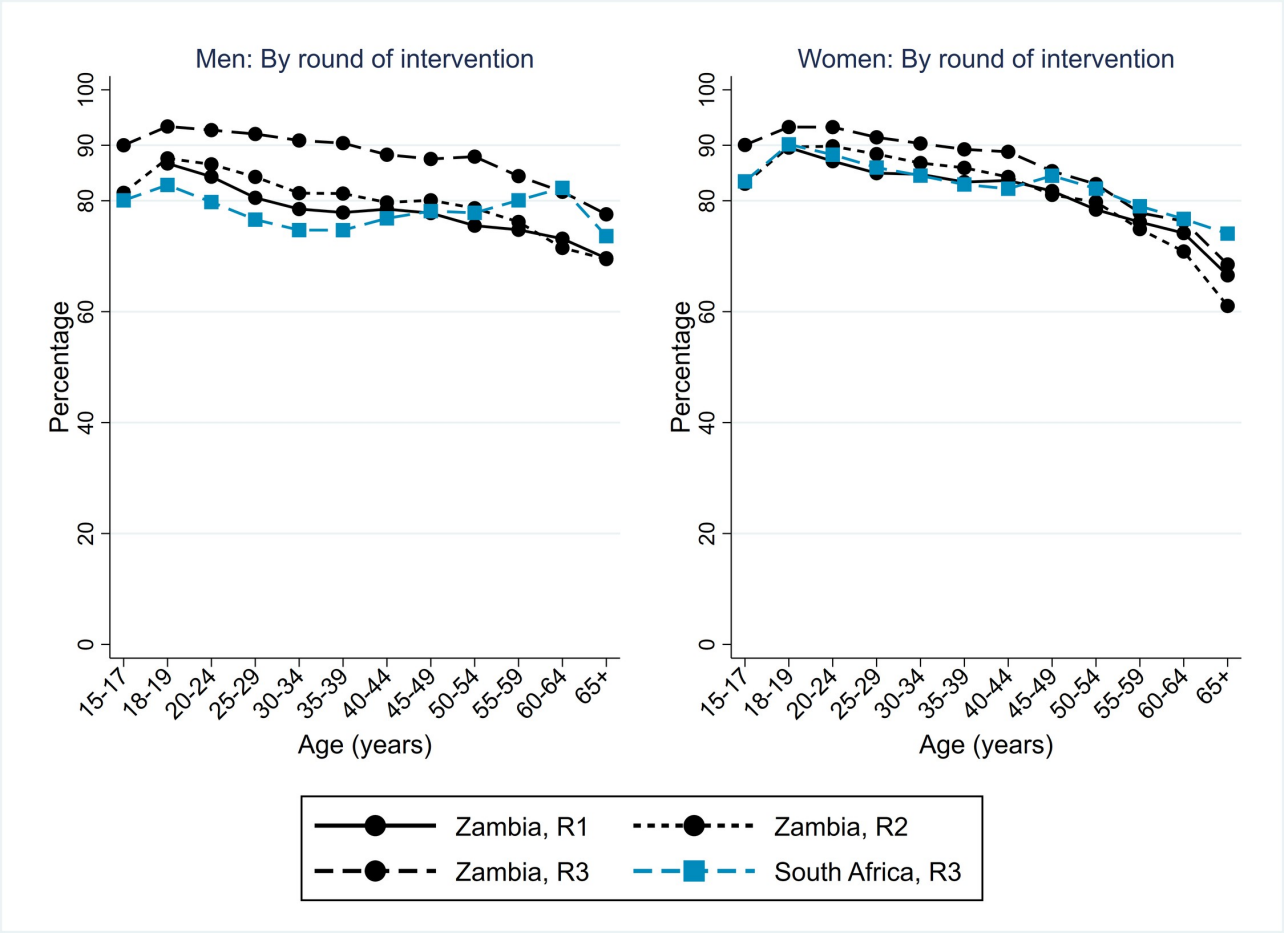
Arm A: Knowledge of HIV status following participation in the PopART intervention, Zambian communities R1–R3, and SA communities R3, by sex and age group. R1, Round 1; R2, Round 2; R3, Round 3; SA, South Africa.

In Zambian communities, knowledge of HIV status following participation in the intervention was very high in R3, at 90% for both men (37,330/41,332) and women (50,432/56,345). It was higher than in R2, following which 83% of men and 86% of women knew their HIV status, due to higher uptake of the offer of HIV testing in R3 (Fig 3). In R3, it was high across the age range 15–54 years for men and 15–44 years for women, and for individuals in all categories of prior participation and residency except for those who had previously participated in the intervention but never previously accepted the offer of HIV testing (S2 Fig).

In SA communities, knowledge of HIV status following participation in the intervention in R3 was also high, though lower than in Zambian communities, at 78% (13,815/17,813) for men and 85% (20,718/24,461) for women, with relatively little variation by age group (Fig 3).

### Arm A: Individuals known by the CHiPs to be HIV positive, and individuals newly diagnosed with HIV, among all who participated in the intervention

**Fig 4:**
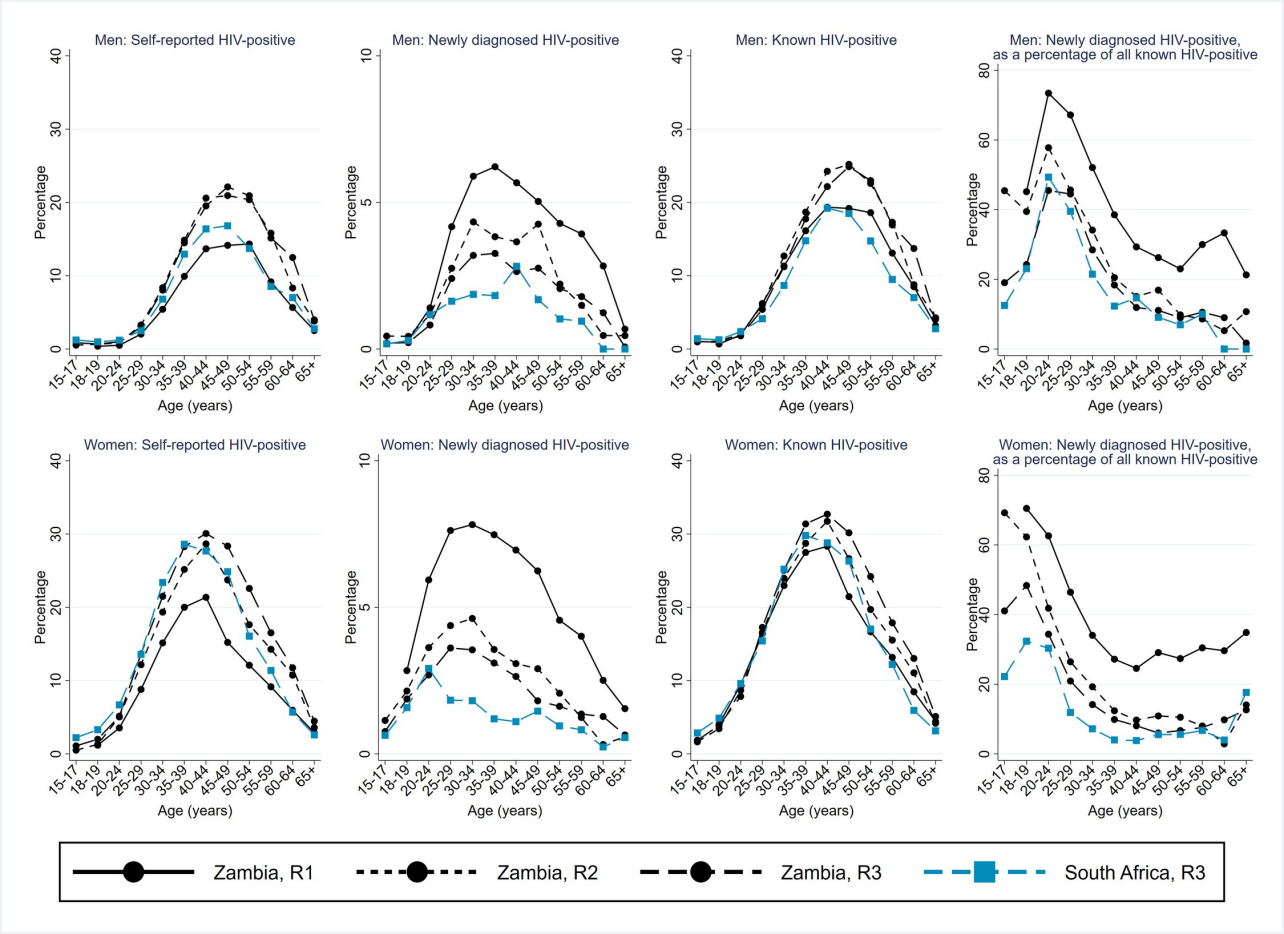
Arm A: Self-report of HIV-positive status, new HIV-positive diagnosis, and total known to be HIV positive, as a percentage of individuals who participated in the intervention; and the percentage of known HIV-positive individuals who were newly diagnosed HIV positive. Zambian communities R1–R3 and SA communities R3, by sex and age group. R1, Round 1; R2, Round 2; R3, Round 3; SA, South Africa.

In Zambian communities, the percentage of men who self-reported they were HIV positive in R3 was similar to R2, both overall at 7.1% and by age group (Fig 4). The percentage who were newly diagnosed with HIV was slightly lower than in R2, at 1.7%. The overall percentage of men who were known to be HIV positive following the R3 visit was 8.8%, slightly lower than following R2.

In SA communities in R3, the percentage of men who self-reported they were HIV positive was 6.7%, while the percentage who were newly diagnosed with HIV was relatively low across the age range and 1.4% overall. The overall percentage of men who were known to be HIV positive following the R3 visit was slightly lower than in Zambian communities, at 8.1%.

For women in Zambian communities, the percentage who self-reported they were HIV positive was slightly higher in R3 than R2, at 13.7%; the percentage who were newly diagnosed with HIV in R3 was slightly lower in R3 than R2, at 2.6%; and the overall percentage who were known to be HIV positive was slightly higher in R3 than R2, at 16.3% (Fig 4). The pattern in SA communities in R3 was similar to this, with overall 17.4% of women known to be HIV positive following R3.

The percentage of “known HIV-positive” individuals who were newly diagnosed was much higher in younger than older individuals in both Zambian and SA communities, peaking among 20- to 29-year-old men and 15- to 24-year-old women (Fig 4).

### Arm A: Estimated HIV prevalence among participants and the total population

Our estimates of HIV prevalence among participants of R3 followed the same age pattern as for “known HIV-positive” prevalence and were slightly higher (S3 Fig). With extrapolation to the total population (and thus its age structure), among men overall HIV prevalence was slightly higher than among participants, whereas among women it was slightly lower.

Age-specific estimates of HIV prevalence, among participants and with extrapolation to the total population, were slightly higher in R2 and R3 in Zambian communities, compared with R1, but broadly the estimates were consistent across rounds and followed the same age pattern. The peak HIV prevalence was among men aged 40 to 49 years (approximately 21%) and women aged 35 to 44 years (approximately 31%), across rounds in Zambian communities and in R3 in SA communities.

These estimates of HIV prevalence were used to calculate the denominator for estimates of coverage against the first 90 target, at the start and end of each round.

### Arm A: Knowledge of HIV-positive status (“first 90”) among participants, and the total population (“central” estimates)

**Fig 5:**
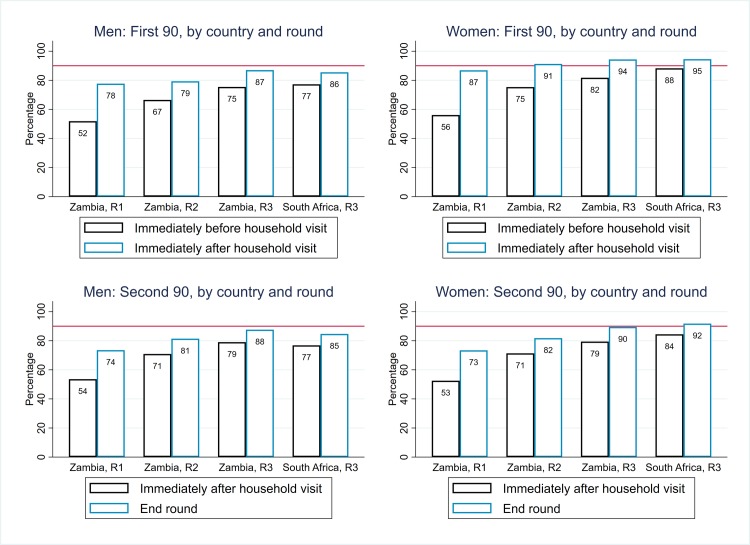
Arm A: Estimates of coverage against the first and second 90 targets, by sex, country, and round (with extrapolation to the total population). R1, Round 1; R2, Round 2; R3, Round 3.

**Fig 6:**
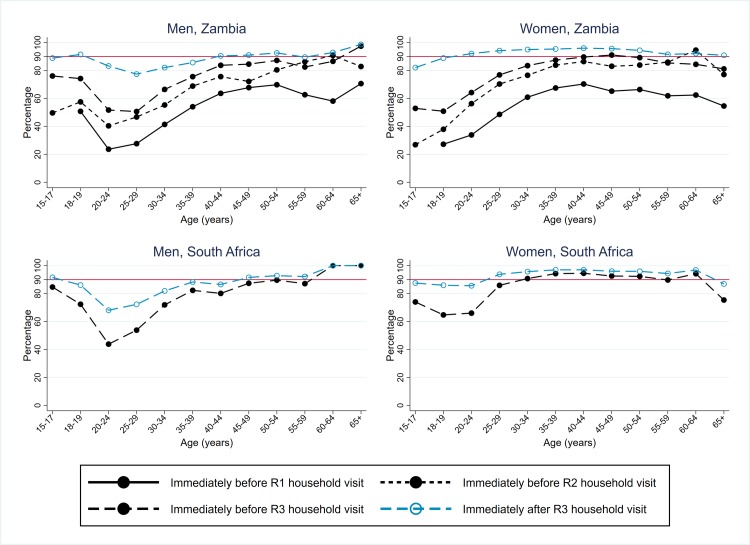
Arm A: Estimates of coverage against the first 90 target, Zambian communities R1–R3, and SA communities R3, by sex and age group (with extrapolation to the total population). R1, Round 1; R2, Round 2; R3, Round 3; SA, South Africa.

In Zambian communities, the estimated percentage of HIV-positive individuals who knew their HIV-positive status progressively increased across R1–R3 (Table 2). Among individuals who participated in the intervention in a particular round, the overall percentage of HIV-positive men who knew their HIV-positive status at the start of the round increased from 52% in R1 to 78% at the start of R3, and by the end of R3 to 97%; figures for women were slightly higher (Table 2, S4 Fig). With extrapolation to the total population, the “start-of-round” estimates were similar to those among participants, but the “end-of-round” estimates were lower. By the end of R3 in Zambian communities, an estimated 87% of HIV-positive men and 94% of HIV-positive women knew their HIV-positive status (Fig 5), 92% overall, and the first 90 target was met or close to being met across the age range (Fig 6).

**Table 2:** Arm A: Estimates of the percentage of HIV-positive individuals who knew their HIV-positive status (first 90), the percentage on ART among individuals who knew their HIV-positive status (second 90), and the percentage on ART among all HIV-positive individuals (ART coverage).

	Estimated HIV+ individuals/population		First 90 (%)		Second 90 (%)		ART coverage (%)	
	*n*/*N*	%	Immediately before annual round visit	Immediately after annual round visit	Immediately after annual round visit	By end of round	Immediately before annual round visit	By end of round
**Participants in the intervention**								
**Zambia, men**								
R1	4,662/45,399	10.3	**52**	89	**47**	72	**42**	64
R2	3,705/35,888	10.3	**71**	94	**64**	80	**60**	75
R3	3,736/41,332	9.0	**78**	**97**	**73**	**85**	**71**	**83**
**Zambia, women**								
R1	9,499/55,703	17.1	**56**	92	**50**	72	**45**	66
R2	8,515/52,210	16.3	**76**	96	**69**	81	**65**	77
R3	9,395/56,345	16.7	**82**	**97**	**77**	**89**	**75**	**87**
**SA**								
Men, R3	1,557/17,813	8.7	77	93	70	84	65	78
Women, R3	4,366/24,461	17.8	88	97	82	92	80	90
**Extrapolated to total population**								
**Zambia, men**								
R1	6,649/61,606	10.8	**52**	78	**54**	74	**42**	57
R2	6,521/61,332	10.6	**67**	79	**71**	81	**56**	65
R3	6,244/64,704	9.7	**75**	**87**	**79**	**88**	**69**	**76**
**Zambia, women**								
R1	11,037/64,305	17.2	**56**	87	**53**	73	**46**	64
R2	10,690/66,106	16.2	**75**	91	**71**	82	**65**	75
R3	11,418/69,458	16.4	**82**	**94**	**79**	**90**	**75**	**84**
**SA**								
Men, R3	3,088/34,245	9.0	**77**	**86**	**77**	**85**	**66**	**72**
Women, R3	6,443/36,859	17.5	**88**	**95**	**84**	**92**	**80**	**87**

Figures in bold are those to which particular attention is drawn in the text.

**Abbreviations:** ART, antiretroviral therapy; R1, Round 1; R2, Round 2; R3, Round 3

Progress toward the first 90 target across R1–R3 in Zambian communities was slowed by the high level of in-migration to, and out-migration from, each CHiP zone (Table 1), which resulted in “start-of-round” estimates of coverage being lower than what had been estimated for the end of the previous round (Fig 5). Among men who were newly resident in the zone in which they were living in R3, overall an estimated 57% knew their HIV-positive status at the start of the round compared with 91% among those who participated in the intervention in R1 and/or R2 and 66% among those who were previously resident in the same zone but did not participate in the intervention in R1 or R2 (Table 3). The corresponding figures among women were 65%, 91%, and 77%. These overall differences were also seen across the age range (S5 Fig).

**Table 3:** Arm A, Zambian communities R3: Estimates of the percentage of HIV-positive individuals who knew their HIV-positive status (first 90), the percentage on ART among individuals who knew their HIV-positive status (second 90), and the percentage on ART among all HIV-positive individuals (ART coverage), by prior residency and prior participation in the intervention in R1–R2.

	Estimated HIV+ individuals/population		First 90 (%)		Second 90 (%)		ART coverage (%)	
	*n*/*N*	%	Immediately before annual round visit	Immediately after annual round visit	Immediately after annual round visit	By end of round	Immediately before annual round visit	By end of round
**Participants in the intervention**								
**Men**								
Participated in R1 and/or R2	2,276/23,308	9.8	91	99	83	89	81	88
Resident in R1 and/or R2, did not participate in either round	261/3,669	7.1	67	95	65	79	61	75
Newly resident in CHiP zone in R3	1,199/14,355	8.4	56	96	55	79	53	76
**Women**								
Participated in R1 and/or R2	6,079/34,547	17.6	92	98	84	91	82	89
Resident in R1 and/or R2, did not participate in either round	211/1,963	10.7	76	95	77	89	73	85
Newly resident in CHiP zone in R3	3,105/19,835	15.7	64	96	64	84	61	81
**Extrapolated to total population**								
**Men**								
Participated in R1 and/or R2	3,173/31,273	10.1	**91**	96	**84**	89	**81**	86
Resident in R1 and/or R2, did not participate in either round	771/9,236	8.3	**66**	76	**81**	87	**62**	66
Newly resident in CHiP zone in R3	2,300/24,195	9.5	**57**	78	**69**	85	**54**	66
**Women**								
Participated in R1 and/or R2	7,091/40,841	17.4	**91**	97	**85**	91	**82**	88
Resident in R1 and/or R2, did not participate in either round	450/3,941	11.4	**77**	86	**87**	93	**74**	80
Newly resident in CHiP zone in R3	3,877/24,676	15.7	**65**	90	**69**	86	**62**	78

Figures in bold are those to which particular attention is drawn in the text.

**Abbreviations:** ART, antiretroviral therapy; CHiP, community HIV care provider; R1, Round 1; R2, Round 2; R3, Round 3

In SA communities, coverage against the first 90 target was high at the start of R3, with overall estimates of 77% for HIV-positive men and 88% for HIV-positive women, and higher by the end of R3 with overall estimates of 86% for HIV-positive men and 95% for HIV-positive women (Table 2, Fig 5). Coverage was high across the age range but relatively lower among HIV-positive men aged 20 to 29 years (Fig 6).

### Arm A: ART uptake among individuals who knew their HIV-positive status (“second 90”), among participants and the total population (“central” estimates”)

In Zambian communities, the overall percentage who were on ART among HIV-positive men who knew their HIV-positive status immediately following participation in a particular round increased from 47% in R1 to 73% at the start of R3, and by the end of R3 to 85%; figures for women were approximately 4% higher (Table 2, S6 Fig).

**Fig 7:**
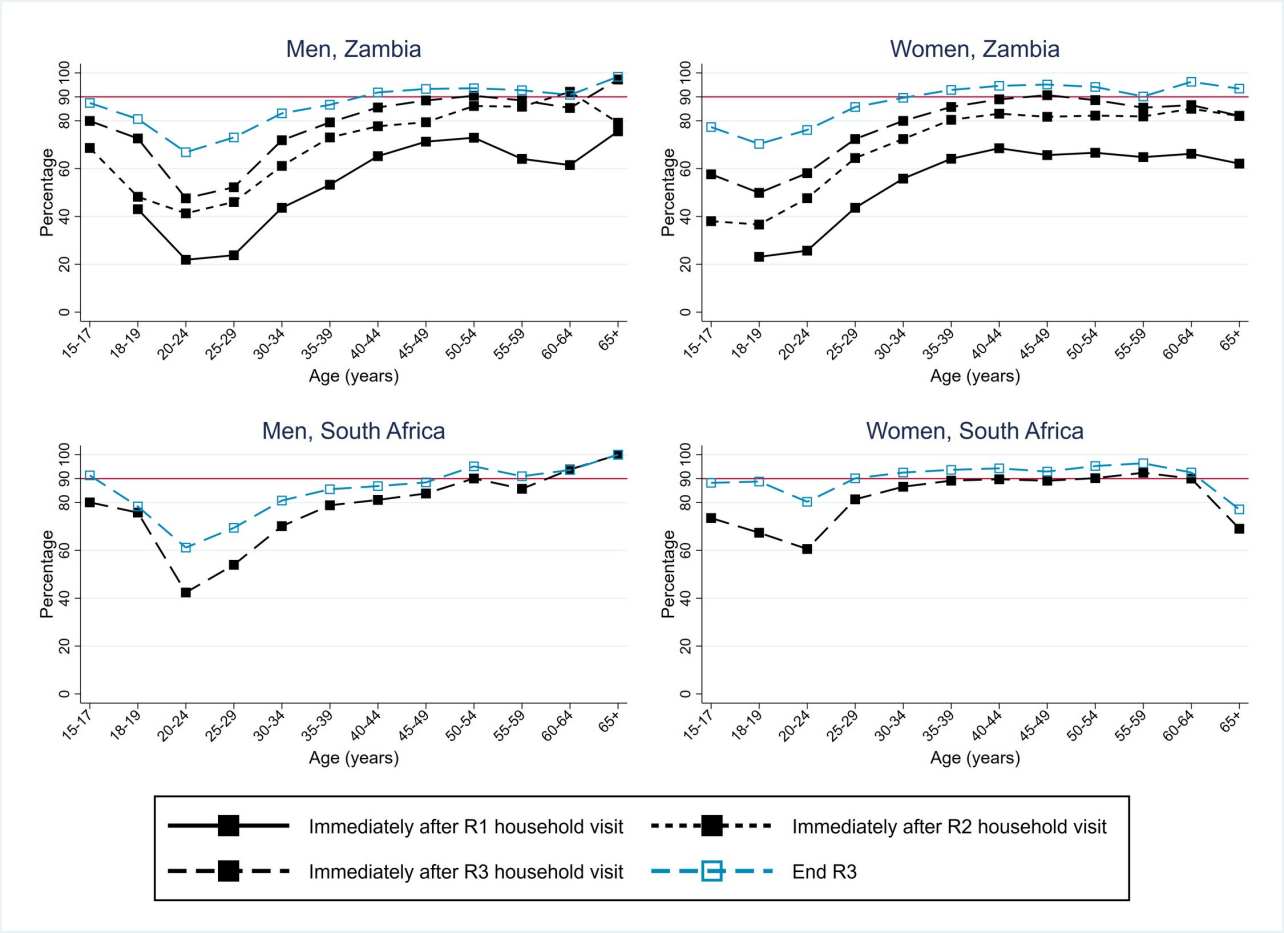
Arm A: Estimates of coverage against the second 90 target, Zambian communities R1–R3, and SA communities R3, by sex and age group (with extrapolation to the total population). R1, Round 1; R2, Round 2; R3, Round 3; SA, South Africa.

With extrapolation to the total population, the “immediately-after-start-of-round” estimates were higher than among participants (due to the conservative assumption that zero nonparticipants were newly diagnosed with HIV during the round, so a new “gap” between knowledge of HIV-positive status and treatment uptake was not created among nonparticipants), and the “end-of-round” estimates were similar. By the end of R3, overall an estimated 88% of men and 90% of women were on ART among those who knew their HIV-positive status (Table 2, Fig 5), and 89% overall. The second 90 target was furthest from being met among younger men (age 18–34 years) and women (age 15–24 years) (Fig 7). As with the first 90, progress toward the second 90 target across R1–R3 in Zambian communities was slowed by the high level of population turnover within each CHiP zone (Table 3).

In SA communities, coverage against the second 90 target was high at the start of R3—with overall estimates of 77% for HIV-positive men and 84% for HIV-positive women—and higher by the end of R3 with overall estimates of 85% for HIV-positive men and 92% for HIV-positive women (Table 2, Fig 5). Coverage was high across the age range but relatively lower among HIV-positive men aged 18 to 34 years (Fig 7).

### Arm A: ART coverage in the total population of HIV-positive individuals (“central” estimates)

There was a progressive increase in estimated ART coverage among all HIV-positive individuals in the population (regardless of knowledge of their HIV-positive status) in Zambian communities across 3 rounds of intervention (Table 2). Among HIV-positive men, estimated ART coverage increased from 42% at the start of R1 to 69% at the start of R3, and to 76% by the end of R3. Among HIV-positive women, estimated ART coverage increased from 46% at the start of R1 to 75% at the start of R3, and to 84% by the end of R3. Overall, across HIV-positive men and women, estimated ART coverage was 82% by the end of R3.

Population turnover slowed the increase in ART coverage across rounds in Zambian communities (Table 3). For example, at the start of R3, an estimated 54% of HIV-positive men were on ART among those who were newly resident in the zone in which they were living in R3. This compared with 81% among those who participated in the intervention in R1 and/or R2, and 62% among those who were previously resident but had not previously participated in the intervention (Table 3).

In SA communities, estimated ART coverage was 72% in HIV-positive men (slightly lower than in Zambian communities) and 87% in HIV-positive women (slightly higher than in Zambian communities) by the end of R3 (Table 2) and overall was 82%.

### Arm A, R3: Sensitivity analyses of estimates of knowledge of HIV-positive status and ART uptake in total population

Estimates of ART coverage, and of coverage against the first and second 90 targets, were not sensitive to different assumptions about individuals who participated in the PopART intervention but whose HIV status was not known to the CHiPs (Table 4). However, they were sensitive to different assumptions about individuals who were resident but did not participate in the intervention during R3; ART coverage estimates were approximately 9% to 15% lower among men and approximately 5% to 13% lower among women, in “conservative” sensitivity analyses that assumed lower testing and treatment coverage among individuals who did not participate in the PopART intervention compared with our “central” assumptions (Table 4).

**Table 4:** Arm A, R3: Sensitivity analysis of estimates of ART coverage and estimates of coverage against the first and second 90 targets: Example “conservative” scenarios.

	**Men**											
	**Zambia**						**SA**					
	**First 90 (%)**		**Second 90 (%)**		**ART coverage (%)**		**First 90 (%)**		**Second 90 (%)**		**ART coverage (%)**	
	Immediately before annual round	Immediately after annual round	Immediately after annual round	By end of round	Immediately before annual round	By end of round	Immediately before annual round	Immediately after annual round	Immediately after annual round	By end of round	Immediately before annual round	By end of round
**Central estimate**	75	87	79	88	69	76	77	86	77	85	66	72
**Scenario 1**^**1**^	75	86	79	87	67	75	77	85	75	82	64	70
**Scenario 2**^**2**^	71	82	72	81	60	67	70	77	67	76	52	58
**Scenario 3**^**3**^	70	80	72	81	58	65	69	76	67	75	51	57
	**Women**											
	**Zambia**						**SA**					
	**First 90 (%)**		**Second 90 (%)**		**ART coverage (%)**		**First 90 (%)**		**Second 90 (%)**		**ART coverage (%)**	
	Immediately before annual round	Immediately after annual round	Immediately after annual round	By end of round	Immediately before annual round	By end of round	Immediately before annual round	Immediately after annual round	Immediately after annual round	By end of round	Immediately before annual round	By end of round
**Central estimate**	82	94	79	90	75	84	88	95	84	92	80	87
**Scenario 1**^**1**^	81	93	79	89	74	83	88	94	84	91	79	86
**Scenario 2**^**2**^	80	92	76	86	70	79	83	89	79	86	70	76
**Scenario 3**^**3**^	79	91	76	86	67	78	82	87	78	85	68	74

^1^Scenario 1 = Among individuals who participated in the intervention in R3 but whose HIV status was not known to the CHiPs: HIV prevalence is twice as high as among individuals who accepted the offer of HIV testing in R3, their knowledge of their HIV-positive status is half the “immediately prior to R3” value among individuals whose HIV-positive status is known to CHIPs following R3, and their ART uptake is half the “immediately prior to R3” value among individuals who self-reported they were HIV positive in R3.

^2^Scenario 2 = As for Scenario 1 for participants. For nonparticipants: HIV prevalence is assumed to be the same as among participants, knowledge of HIV-positive status immediately prior to R3 is 0.8 times the value for participants, and ART uptake among individuals who know their HIV-positive status is 0.8 times the value for participants.

^3^Scenario 3 = As for Scenario 2, except that HIV prevalence is assumed to be 1.25 times the value for participants. This is the “worst-case” scenario, among those considered in our sensitivity analyses.

All assumptions are made within strata defined by every combination of sex, community, age group, and (for Zambia) prior residency and participation in the intervention.

Abbreviations: ART, antiretroviral therapy; CHiP, community HIV care provider; R3, Round 3

### Arm A: Time to ART initiation after CHiP referral to HIV care

**Fig 8:**
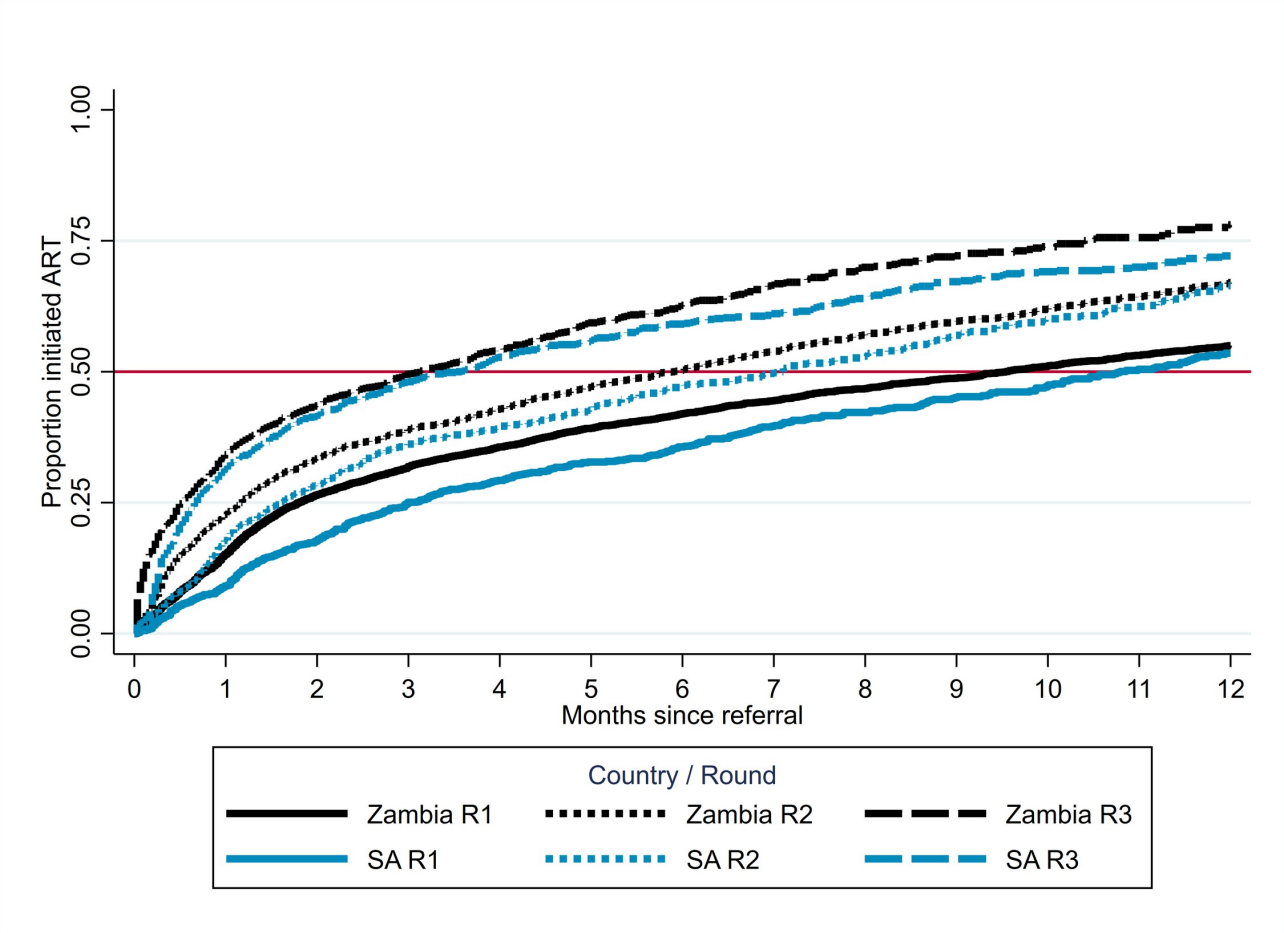
Arm A: Time from CHiP referral to ART initiation among individuals referred to HIV care, by country and round of referral. ART, antiretroviral therapy; CHiP, community HIV care provider; R1, Round 1; R2, Round 2; R3, Round 3; SA, South Africa.

In R3, by 31 October 2017, 2,554 HIV-positive individuals had been referred to HIV care in Zambian communities and 928 in SA communities. Most were referred after testing HIV positive in R3–75% in Zambia and 68% in SA—and the rest self-reported they were HIV positive. The median time to ART initiation after referral to HIV care was approximately 3 months in both countries, considerably shorter than in previous rounds (*P* < 0.001), and by 12 months after referral an estimated approximately 70% had initiated ART (Fig 8). The shortening of the time to ART initiation over R1–R3 was seen overall, and for both men and women [17].

### Comparison of Arm A with Arm B communities

Overall, and with stratification by country and round and sex and age group, all measures of intervention coverage were similar in Arm A and Arm B communities (S7–S12 Figs show the equivalent figures to those provided for Arm A in Figs 2–4 and 6–8).

**Fig 9:**
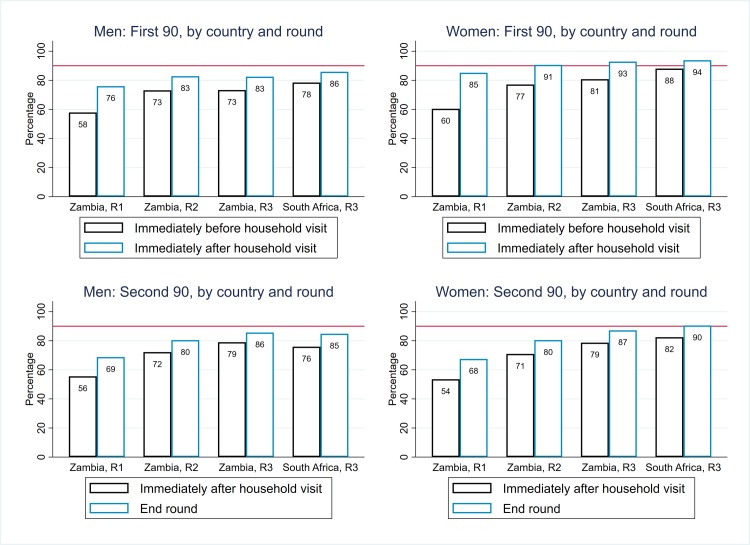
Arm B: Estimates of coverage against the first and second 90 targets, by sex, country, and round (with extrapolation to the total population). R1, Round 1; R2, Round 2; R3, Round 3.

**Fig 10:**
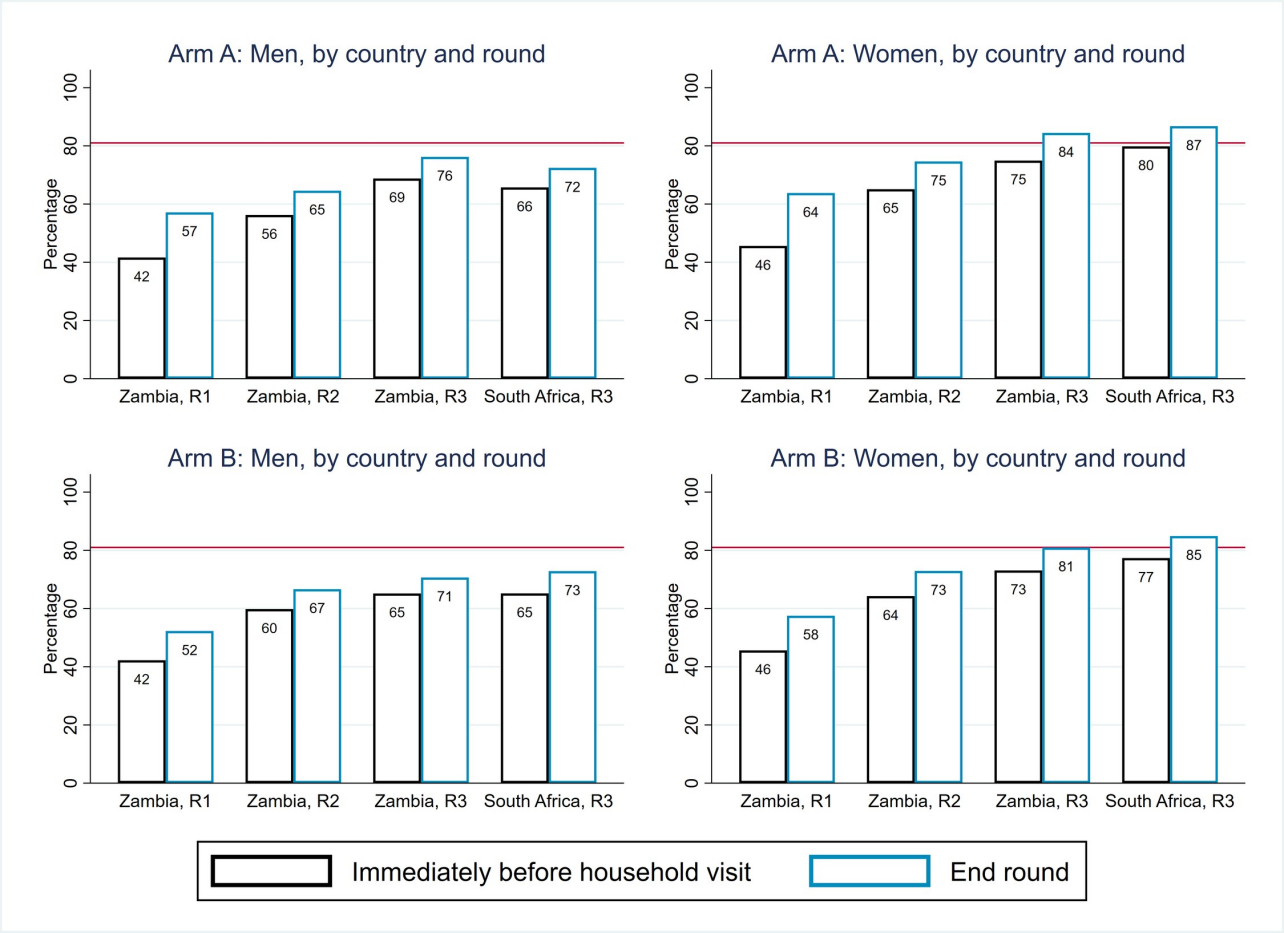
Arm A and Arm B: ART coverage estimates, by trial arm, sex, country, and round (with extrapolation to the total population). ART, antiretroviral therapy; R1, Round 1; R2, Round 2; R3, Round 3.

Coverage against the first and second 90 targets in Arm B is summarised in Fig 9, for direct comparison with Arm A as summarised in Fig 5. In Fig 10, ART coverage estimates for Arm A and Arm B are compared by country, round, and sex; if anything, coverage was slightly lower in Arm B than in Arm A, but the 2 trial arms had similar trajectories across rounds in Zambian communities and similar end-of-intervention coverage in SA communities. A formal comparison between Arm A and Arm B for these 3 coverage measures at the end of R3—based on 14 community-level summaries and applying the approach to trial arm comparisons that was set out in the HPTN 071 statistical analysis plan [15]—showed that there was no statistical evidence of a difference between the 2 trial arms (S1, S2 and S3 Tables).

### Estimates of coverage against the third 90 target from the population cohort study

Estimates of coverage against the third 90 target were at or close to 90% in Zambian communities, across men and women and Arm A and Arm B (Table 5). In SA communities, coverage against the 3^rd^ 90 target was approximately 85% in women and in the range of approximately 80% to 90% for men. Overall, estimates were similar in Arm A and Arm B.

**Table 5:** Viral suppression among HIV-positive individuals who self-reported they had taken ART in the previous 1 month, from the 24-month follow-up (mid-2016 to mid-2017) of the population cohort study.

	Arm A		Arm B	
	*n*/*N*^1^	%	*n*/*N*^1^	%
Zambia, men	84/98	85.7	80/89	89.9
Zambia, women	615/674	91.2	608/669	90.9
SA, men	25/28	89.3	31/39	79.5
SA, women	286/337	84.9	308/364	84.6

^1^*n*/*N* = number virally suppressed/total HIV-positive and self-reported taking ART in the previous 1 month.

**Abbreviation:** ART, antiretroviral therapy

## Discussion

The HPTN 071 (PopART) trial, implemented in urban/peri-urban communities in Zambia and SA during 2014–2018, aimed to establish whether universal testing and universal treatment for HIV could reduce population-level HIV incidence in a generalised HIV epidemic. To be effective, the PopART intervention needed to achieve high rates of HIV testing and treatment coverage across the community, and so a key secondary aim of the study was to learn whether this goal could be attained. We found that high levels of HIV testing and treatment coverage were achieved in communities that received the PopART UTT intervention, especially among women, and the cumulative 90–90 target of 81% ART coverage was met overall. This took 4 years, to the end of the study, by which time approximately 95% of HIV-positive women and approximately 85% of HIV-positive men knew their HIV status; among these individuals, approximately 90% of women and approximately 85% of men were on ART. Among all HIV-positive individuals, regardless of knowledge of their HIV-positive status, ART coverage was approximately 85% in women and approximately 75% in men in both the Zambian and SA communities, around 10% higher than after the first 2 years of the intervention and up from around 45% at the start of the study.

Progress toward reaching high testing and treatment coverage was slowed by high rates of mobility and in-migration among both men and women, with mobile individuals and in-migrants having lower knowledge of HIV-positive status and ART coverage than “stable” residents, as reported previously [7] and also found in the ANRS 12249 TasP trial [18]. The way in which the PopART intervention was delivered through “rounds” of visits to every household meant that it was effective in identifying and including newly resident individuals, but only with a delay. Progress was also slowed by the relative difficulty of contacting men at home [7,19–23], which persisted throughout the study. Service coverage among men and mobile individuals might have been accelerated if CHiPs had been able to offer services outside of a household setting more often.

The main gap in coverage against the first and second of the 90-90-90 targets that remained after 4 years of intervention was among men aged 18 to 34 years and women aged 15 to 24 years, consistent with findings from the SEARCH and BCPP/YaTsie studies [4,5]. In both groups, coverage progressively increased, and participation in the intervention, testing uptake among participants, and the time from CHiP referral to ART initiation were similar to or higher than among individuals aged 35 to 54 years. The reason for the remaining postintervention gap was that the proportion of HIV-positive individuals who knew their HIV-positive status “pre-intervention” and at the start of each round was much lower for younger than older individuals. This reflected fewer prior opportunities for facility-based HIV testing, relatively high HIV incidence rates compared with HIV prevalence, and relatively high mobility and in-migration rates. The “pre-intervention” gap among younger individuals might be reduced by making facility-based services more “youth friendly,” and steps toward this were taken during the last year of intervention as part of the nested “PopART-for-Youth” (P-ART-Y) study. The provision of HIV self-testing could also contribute [14,24–26].

Participation in the PopART intervention, and the uptake of HIV testing among participants, increased between the second and third rounds in Zambia. This showed that service uptake can improve with time and accrued experience, rather than worsen with fatigue, when a community increasingly values the services that are being offered and trusts the individuals who are providing them. On the other hand, a proportion of individuals never participated in the intervention despite CHiPs being continuously present in the community for 4 years, and even among participants a proportion repeatedly declined the offer of HIV testing. These factors placed an upper limit on the testing and treatment coverage that could be achieved. In the future, it is possible that more service provision in the community outside a household setting and the option of HIV self-testing might reach some previously unreached individuals [14,24–27].

In the first round of intervention, the time between referral of an HIV-positive individual to HIV care by CHiPs and ART initiation was much slower than originally targeted, slowing progress toward high treatment coverage. However, by the third round of intervention, the median time from CHiP referral to ART initiation had been shortened from approximately 10 months in the first round to approximately 3 months. This reflected increased trust in the CHiPs with time, as well as the success of the various strategies that were implemented to facilitate linkage to HIV care—tailored according to the needs of each individual—during the entirety of the second and third rounds of intervention. At the same time, it confirmed earlier findings that a proportion of HIV-positive individuals need and/or wish to take a period of time from when they first learn their HIV-positive status to when they link to HIV care [8].

Our finding that the first and second of the 90-90-90 targets can be attained through several years of community-wide delivery of a combination HIV prevention package that includes universal testing and facilitated linkage to universal treatment is consistent with previously reported findings [4,5]. In the SEARCH study in rural Uganda and Kenya, HIV testing services were delivered annually from a central community location for a period of several weeks, supplemented by household visits. In the BCPP/YaTsie study in Botswana, door-to-door HIV testing services were delivered annually, supplemented with community provision in a nonhousehold setting. Other studies have also demonstrated the potential of community-wide delivery of home-based testing and support for linkage to care to achieve high testing and treatment coverage prior to guidelines changing to recommend universal ART, for example, in 2 communities in Uganda and SA [28]. At a national level, as of 2017, Malawi and eSwatini had come close to achieving the 90-90-90 targets through the expansion of community-based HIV testing services alongside facility-based testing at multiple clinical service points, as well as strengthened support for linkage to HIV care and ART initiation following testing [29–31]. Taken in this context, our study extends the generalisability of the finding that 90-90-90 can be achieved through persistent delivery of UTT services, in particular to urban settings and to 2 additional countries.

There were several limitations to our study. First, our estimates of testing and treatment coverage for all men (as opposed to men who participated in the PopART intervention) relied on considerable extrapolation because in each round approximately one-third of men did not participate. We may have overestimated what was achieved for ART coverage among men by up to around 10% in absolute terms. Second, our findings are for a service delivery model that was relatively intensive, with 2 CHiPs for every 500 households and approximately 1,000 to 1,500 adults. Although the cost per resident aged ≥15 years was relatively modest at approximately $6 to $8 per year, the PopART intervention might need to be adapted and/or streamlined to be more affordable for programmatic delivery on a large scale. Third, we did not have comparable data on testing and treatment coverage from the “standard-of-care” trial arm (Arm C), because in Arm C communities, there were no CHiPs delivering the PopART intervention. The trial population cohort study was conducted in all 21 study communities but could not provide unbiased estimates of testing and treatment coverage because all participants were offered rapid HIV testing at every follow-up survey. Additionally, information on viral suppression was not collected by CHiPs as part of intervention service delivery, and for most of the study period, viral load testing was not routine in the study community clinics in Zambia. Although it was a limitation of our data that ART uptake was self-reported, a strength was that, among those who self-reported that they were on ART, a high percentage (approximately 80%) showed their ART card to CHiPs.

While we focused our attention on analysis of findings from the “full” PopART UTT intervention arm over 4 years of intervention delivery, we also showed that achievements were similar in the “intermediate” intervention arm. At trial start, the 2 trial arms differed only in the CD4 threshold used for ART eligibility; within 6–12 months of study start, the difference diminished when the CD4 threshold for ART eligibility was raised to 500 cells/mm^3^ in the intermediate intervention arm, and it was then eliminated when universal ART became standard of care during the last 18 months of intervention delivery. This meant that the 2 trial arms were very similar for ART eligibility for most of the 4-year intervention-delivery period, and the time when they differed most—the first year of the study—was also when the time from CHiP referral to linkage to HIV care was relatively slow across all study communities. From our comparison of testing and treatment coverage in Arms A and B, there was no evidence of a differential that could explain the larger reduction in HIV incidence that was observed in Arm B compared with Arm A communities. Other possible explanations being explored include differentials in migration, partners from outside the study community, and behaviour change.

## Conclusion

Our study showed that very high HIV testing and treatment coverage can be achieved over several years through persistent delivery of universal testing, facilitated linkage to HIV care, and universal treatment services. Our findings are consistent with previously reported findings from southern and east Africa, extending their generalisability to urban settings with high rates of in-migration and mobility and to Zambia and SA.

## Supporting information

S1 STROBE Checklist STROBE, strengthening the reporting of observational studies in epidemiology. (DOC)

S1 Fig Arm A: Participation in the PopART intervention in Zambia in R3, by previous participation in the intervention and prior residency in the same area of the community, and by sex and age group. (TIF)

S2 Fig Arm A: Knowledge of HIV status following participation in the PopART intervention in Zambia in R3, by previous participation in the intervention and prior residency in the same area of the community, and by sex and age group. (TIF)

S3 Fig Arm A: Individuals known to be HIV positive following participation in the intervention, and estimated HIV prevalence among individuals who participated in the intervention and with extrapolation to the total population, Zambia R1–R3 and South Africa R3, by sex and age group. (TIF)

S4 Fig Arm A: Estimates of coverage against the first 90 target, Zambia R1–R3 and South Africa R3, by sex and age group—Among individuals who participated in the intervention. (TIF)

S5 Fig Arm A: Estimates of coverage against the first 90 target, Zambia R3, by sex and age group and previous residency and participation in the intervention in the same CHiP zone—With extrapolation to the total population. (TIF)

S6 Fig Arm A: Estimates of coverage against the second 90 target, Zambia R1–R3 and South Africa R3, by sex and age group—Among individuals who participated in the intervention. (TIF)

S7 Fig Arm B: Participation in the PopART intervention, Zambian communities R1–R3 and SA communities R3, by sex and age group. (TIF)

S8 Fig Arm B: Knowledge of HIV status following participation in the PopART intervention, Zambian communities R1–R3 and SA communities R3, by sex and age group. (TIF)

S9 Fig Arm B: Self-report of HIV-positive status, new HIV-positive diagnosis, and total known to be HIV positive, as a percentage of individuals who participated in the intervention; and the percentage of known HIV-positive individuals who were newly diagnosed HIV positive. Zambian communities R1–R3 and SA communities R3, by sex and age group.
(TIF)

S10 Fig Arm B: Estimates of coverage against the first 90 target, Zambian communities R1–R3 and SA communities R3, by sex and age group—With extrapolation to the total population. (TIF)

S11 Fig Arm B: Estimates of coverage against the second 90 target, Zambian communities R1–R3 and SA communities R3, by sex and age group—With extrapolation to the total population. (TIF)

S12 Fig Arm B: Time from CHiP referral to ART initiation among individuals referred to HIV care, by country and round of referral. (TIF)

S1 Table Estimates of the percentage of HIV-positive individuals who knew their HIV-positive status immediately after the CHiP household visit of R3, among the estimated total population of HIV-positive individuals aged ≥15 years who were resident at the time of the CHiP household visit in R3 (first 90). Comparison of Arm A with Arm B communities, across 7 triplets of communities.
(DOCX)

S2 Table Estimates of the percentage of HIV-positive individuals who were on ART by the end of R3, among the estimated total population of HIV-positive individuals aged ≥15 years who knew their HIV-positive status immediately after the CHIP household visit of R3 and remained resident in the same CHiP zone at the end of R3 (second 90). Comparison of Arm A with Arm B communities, across 7 triplets of communities.
(DOCX)

S3 Table Estimates of the percentage of HIV-positive individuals who were on ART by the end of R3, among the estimated total population of HIV-positive individuals aged ≥15 years who were resident at the time of the CHiP household visit in R3 and remained resident in the same CHiP zone at the end of R3 (ART coverage). Comparison of Arm A with Arm B communities, across 7 triplets of communities.
(DOCX)

S1 Data Aggregate dataset that can be used to replicate the main analyses presented in this paper. (CSV)

S2 Data Data dictionary for the S1 Data. (DOCX)

## Abbreviations

ART: antiretroviral therapy
BCPP: Botswana Combination Prevention Project
CHiP: community HIV care provider
CRT: cluster-randomised trial
DSMB: Data Safety and Monitoring Board
HPTN: HIV Prevention Trials Network
P-ART-Y: PopART-for-Youth study
PHIA: Population-based HIV Impact Assessment
PopART: Population Effects of Antiretroviral Therapy to Reduce HIV Transmission
R1: Round 1
R2: Round 2
R3: Round 3
SEARCH: Sustainable East Africa Research in Community Health trial
STROBE: Strengthening the Reporting of Observational studies in Epidemiology
TasP: Treatment as Prevention trial
UNAIDS: The Joint United Nations Programme on HIV/AIDS
UTT: universal testing and treatment
