# Supplementary material for: HIV testing and treatment coverage achieved after 4 years across 14 urban and peri-urban communities in Zambia and South Africa: An analysis of findings from the HPTN 071 (PopART) trial
Source: PLoS Med. 2020 Apr 2;17(4):e1003067. doi: 10.1371/journal.pmed.1003067 (PMC7117659; doi:10.1371/journal.pmed.1003067)
Supplement: S1 STROBE Checklist — (DOC) [file pmed.1003067.s001.doc]

STROBE Statement—checklist of items that should be included in reports of observational studies

|  | Item No | Recommendation |
| --- | --- | --- |
| **Title and abstract** | 1 | (*a*) Indicate the study’s design with a commonly used term in the title or the abstract  [Title says “findings from the HPTN 071 (PopART) trial”.] |
|  |  | (*b*) Provide in the abstract an informative and balanced summary of what was done and what was found  [What was done is described in the first paragraph of the “Methods and Findings” section. What was found is described in the second paragraph of the “Methods and Findings” section.] |
| Introduction | | |
| Background/rationale | 2 | Explain the scientific background and rationale for the investigation being reported  [See introduction, paragraphs 1-4.] |
| Objectives | 3 | State specific objectives, including any prespecified hypotheses  [See introduction, paragraph 5.] |
| Methods | | |
| Study design | 4 | Present key elements of study design early in the paper  [Provided in paragraph 1 of Methods, section “Setting and trial design”.] |
| Setting | 5 | Describe the setting, locations, and relevant dates, including periods of recruitment, exposure, follow-up, and data collection  [Provided in paragraph 1 of Methods, section “Setting and trial design”, and paragraph 1 of Methods, section “PopART intervention”.] |
| Participants | 6 | (*a*) *Cohort study*—Give the eligibility criteria, and the sources and methods of selection of participants. Describe methods of follow-up  *Case-control study*—Give the eligibility criteria, and the sources and methods of case ascertainment and control selection. Give the rationale for the choice of cases and controls  *Cross-sectional study*—Give the eligibility criteria, and the sources and methods of selection of participants  [Provided in paragraph 1 of section “Setting and trial design”, paragraph 2 of Methods section “PopART intervention” and paragraph 1 of section “Data collection, outcomes, and explanatory variables”.] |
|  |  | (*b*) *Cohort study*—For matched studies, give matching criteria and number of exposed and unexposed  *Case-control study*—For matched studies, give matching criteria and the number of controls per case  [Not applicable] |
| Variables | 7 | Clearly define all outcomes, exposures, predictors, potential confounders, and effect modifiers. Give diagnostic criteria, if applicable  [Provided in Methods, section “Data collection, outcomes and explanatory variables”, paragraphs 1 and 2; Methods, section “Estimates of the “third 90” from the HPTN 071 (PopART) population cohort study”, paragraph 1.] |
| Data sources/ measurement | 8* | For each variable of interest, give sources of data and details of methods of assessment (measurement). Describe comparability of assessment methods if there is more than one group  [Provided in Methods, section “Data collection, outcomes and explanatory variables”, paragraph 1; and Methods, section “Estimation of the number of HIV-positive individuals in the population, and coverage against the first and second 90 targets”, paragraph 1 and corresponding Box 1; and Methods, section “Estimation of the time from CHiP referral to ART initiation”, paragraph 1; and Methods, section “Estimates of the “third 90” from the HPTN 071 (PopART) population cohort study”, paragraph 1.] |
| Bias | 9 | Describe any efforts to address potential sources of bias  [Provided in Methods, section “Estimation of the number of HIV-positive individuals in the population, and coverage against the first and second 90 targets” in Box 1, where sensitivity analyses are summarised.] |
| Study size | 10 | Explain how the study size was arrived at  [Provided in Methods, section “Setting and trial design”, paragraph 1, and Fig 1 that summarises the trial design. Analyses in this manuscript were based on the total population aged ≥15 years in the study communities that were randomised to receive the PopART intervention.] |
| Quantitative variables | 11 | Explain how quantitative variables were handled in the analyses. If applicable, describe which groupings were chosen and why  [Provided in Methods, section “Data collection, outcomes and explanatory variables” and Box 1; and Methods, section “Estimates of the “third 90” from the HPTN 071 (PopART) population cohort study”, paragraph 1.] |
| Statistical methods | 12 | (*a*) Describe all statistical methods, including those used to control for confounding  [Provided in Methods, section “Data collection, outcomes and explanatory variables”, paragraph 1; and Methods, section “Estimation of the number of HIV-positive individuals in the population, and coverage against the first and second 90 targets”, paragraph 1 and corresponding Box 1; and Methods, section “Estimation of the time from CHiP referral to ART initiation”, paragraph 1; and Methods, section “Estimates of the “third 90” from the HPTN 071 (PopART) population cohort study”, paragraph 1.] |
|  |  | (*b*) Describe any methods used to examine subgroups and interactions  [Provided in Methods, section “Data collection, outcomes, and explanatory variables”, paragraph 2.] |
|  |  | (*c*) Explain how missing data were addressed  [Methods for extrapolation from individuals who participated in the intervention to non-participants are explained in Methods, Box 1.] |
|  |  | (*d*) *Cohort study*—If applicable, explain how loss to follow-up was addressed  *Case-control study*—If applicable, explain how matching of cases and controls was addressed  *Cross-sectional study*—If applicable, describe analytical methods taking account of sampling strategy  [Provided in Methods, Box 1; and in Methods, section “Estimation of the time from CHiP referral to ART initiation”, paragraph 1.] |
|  |  | (*e*) Describe any sensitivity analyses  [Provided in Methods, Box 1.] |

Continued on next page

| Results | | |
| --- | --- | --- |
| Participants | 13* | (a) Report numbers of individuals at each stage of study—eg numbers potentially eligible, examined for eligibility, confirmed eligible, included in the study, completing follow-up, and analysed  [Provided in Results, section “Arm A: R3: Households visited and enumerated, total individuals enumerated, and population structure”; and in subsequent sub-sections of the results that report on key outcomes; and in Tables 1-4.] |
|  |  | (b) Give reasons for non-participation at each stage  [Not applicable] |
|  |  | (c) Consider use of a flow diagram  [Not provided, but study population is summarised in Table 1, and key numerators and denominators are provided in the text and tables.] |
| Descriptive data | 14* | (a) Give characteristics of study participants (eg demographic, clinical, social) and information on exposures and potential confounders  [Provided in Table 1.] |
|  |  | (b) Indicate number of participants with missing data for each variable of interest  [Not applicable; but methods of extrapolation from participants in the PopART intervention to non-participants are provided in Methods, Box 1.] |
|  |  | (c) *Cohort study*—Summarise follow-up time (eg, average and total amount)  [Not applicable] |
| Outcome data | 15* | *Cohort study*—Report numbers of outcome events or summary measures over time |
|  |  | *Case-control study—*Report numbers in each exposure category, or summary measures of exposure |
|  |  | *Cross-sectional study—*Report numbers of outcome events or summary measures  [Data on the numbers with the outcome, and summary measures, are provided in the Results text, tables, and figures.] |
| Main results | 16 | (*a*) Give unadjusted estimates and, if applicable, confounder-adjusted estimates and their precision (eg, 95% confidence interval). Make clear which confounders were adjusted for and why they were included  [Results for the key variables of interest are provided throughout the Results text, and in Figures 2-10 and Tables 1-4.] |
|  |  | (*b*) Report category boundaries when continuous variables were categorized  [Not applicable] |
|  |  | (*c*) If relevant, consider translating estimates of relative risk into absolute risk for a meaningful time period  [For Results, section “Estimates of the time from CHiP referral to ART initiation” we have provided absolute measures in terms of the percentage who initiated ART by a given time point after referral, and also shown this “absolute” measure in Figure 8.] |
| Other analyses | 17 | Report other analyses done—eg analyses of subgroups and interactions, and sensitivity analyses  [Key analyses are disaggregated by trial arm, country, round of intervention; all except estimates of time from CHiP referral to ART initiation are disaggregated by sex; and most are further disaggregated by age group. Estimates of coverage against the first and second 90 targets, and ART coverage, are disaggregated according to whether an individual was newly or previously resident, and newly or previously participated in the PopART intervention (Table 3). Sensitivity analyses are summarised in Table 4, with corresponding text in Results, section “Arm A, R3: Sensitivity analyses of estimates of knowledge of HIV status and ART uptake in total population”.] |
| Discussion | | |
| Key results | 18 | Summarise key results with reference to study objectives  [Provided in Discussion, paragraph 1] |
| Limitations | 19 | Discuss limitations of the study, taking into account sources of potential bias or imprecision. Discuss both direction and magnitude of any potential bias  [Provided in Discussion, paragraph 7] |
| Interpretation | 20 | Give a cautious overall interpretation of results considering objectives, limitations, multiplicity of analyses, results from similar studies, and other relevant evidence  [Provided in Discussion, paragraphs 2, 3, 4, 5, 6, and 8] |
| Generalisability | 21 | Discuss the generalisability (external validity) of the study results  [Provided in Discussion, paragraph 6 and the Conclusion] |
| Other information | | |
| Funding | 22 | Give the source of funding and the role of the funders for the present study and, if applicable, for the original study on which the present article is based  [A statement on this was provided as part of the manuscript submission] |

*Give information separately for cases and controls in case-control studies and, if applicable, for exposed and unexposed groups in cohort and cross-sectional studies.

**Note:** An Explanation and Elaboration article discusses each checklist item and gives methodological background and published examples of transparent reporting. The STROBE checklist is best used in conjunction with this article (freely available on the Web sites of PLoS Medicine at http://www.plosmedicine.org/, Annals of Internal Medicine at http://www.annals.org/, and Epidemiology at http://www.epidem.com/). Information on the STROBE Initiative is available at www.strobe-statement.org.
