# Supplementary figures and images for: HIV testing and treatment coverage achieved after 4 years across 14 urban and peri-urban communities in Zambia and South Africa: An analysis of findings from the HPTN 071 (PopART) trial
Source: PLoS Med. 2020 Apr 2;17(4):e1003067. doi: 10.1371/journal.pmed.1003067 (PMC7117659; doi:10.1371/journal.pmed.1003067)

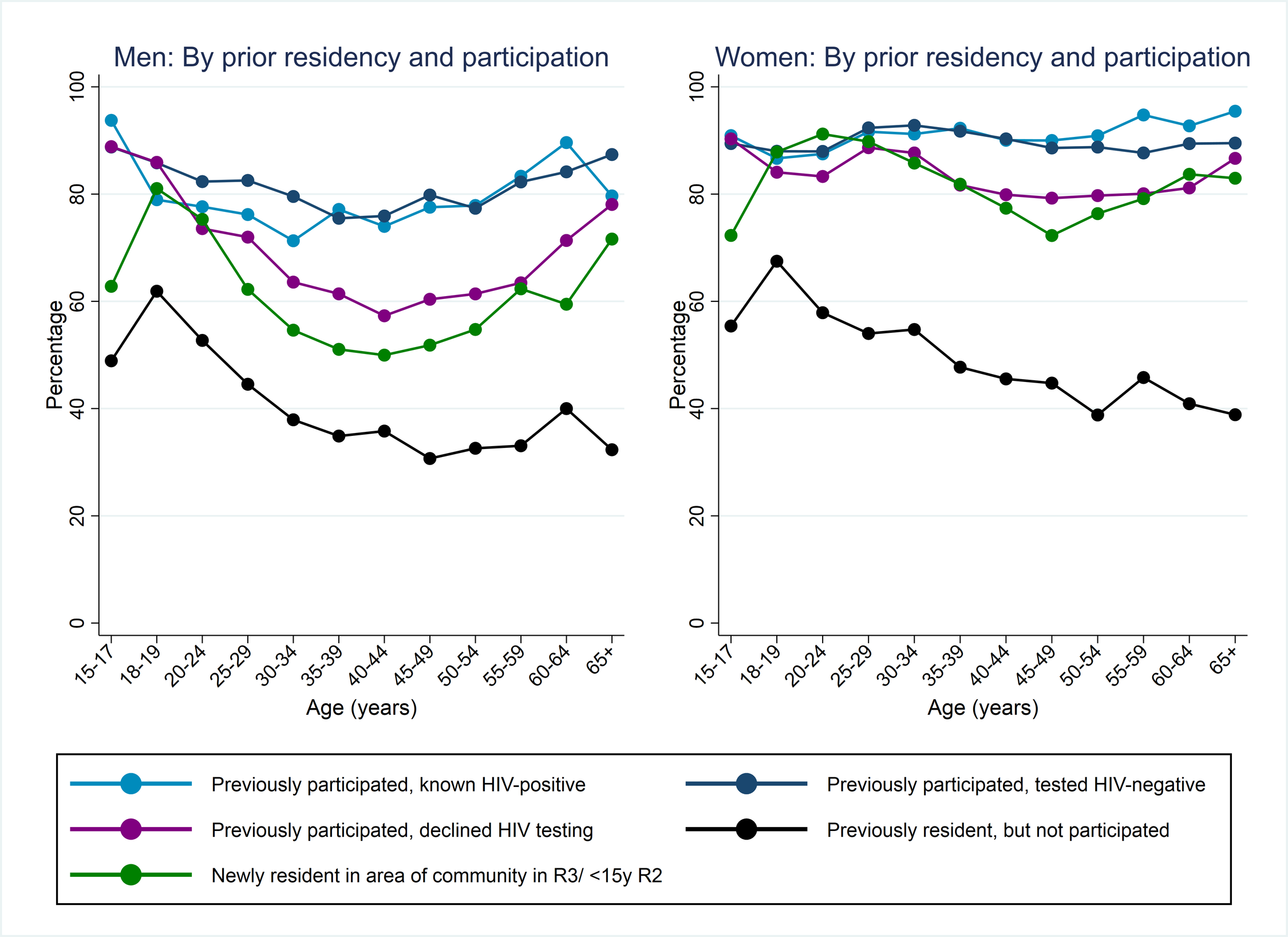

Supplement: S1 Fig — (TIF) [file pmed.1003067.s002.tif]

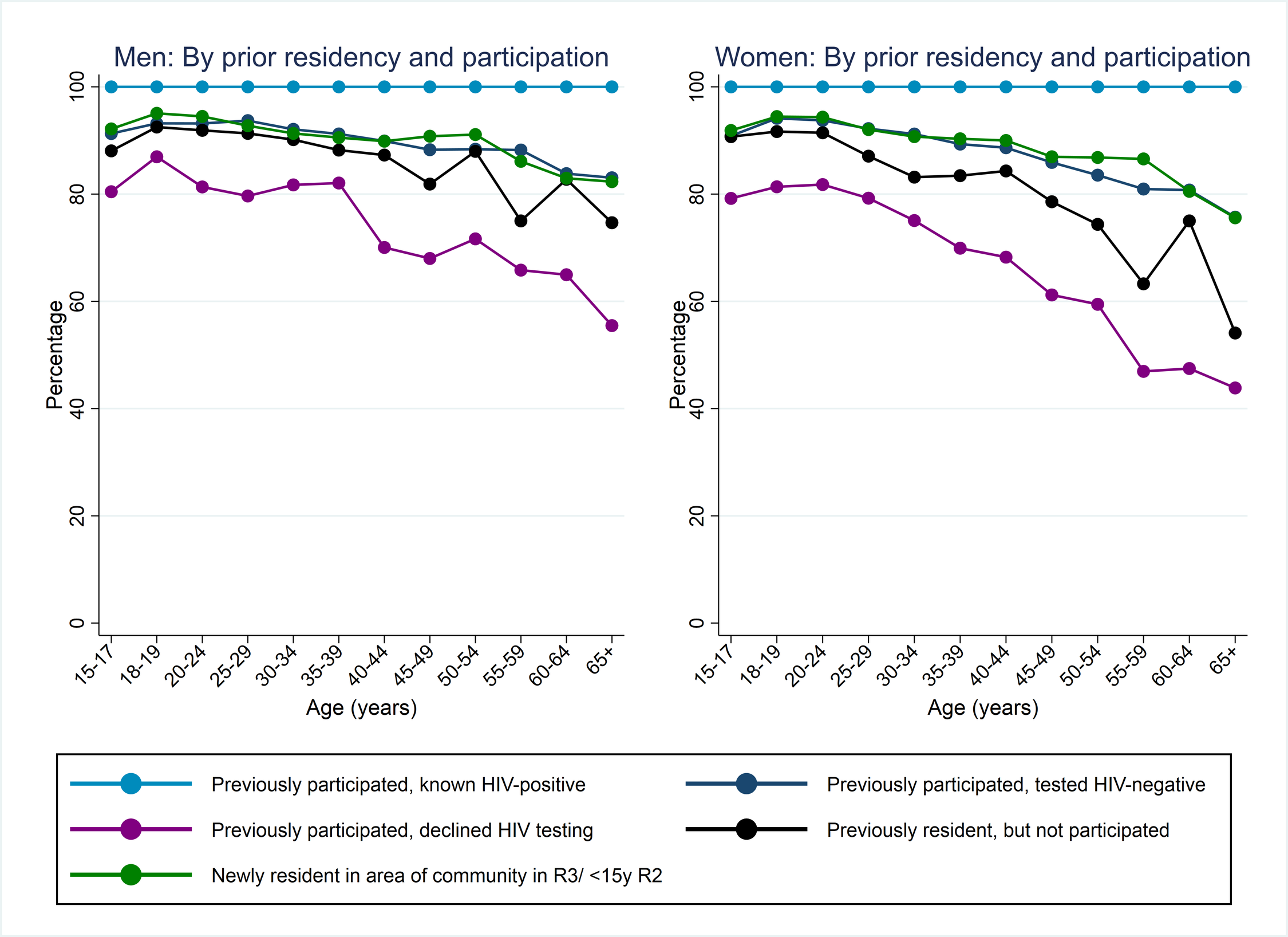

Supplement: S2 Fig — (TIF) [file pmed.1003067.s003.tif]

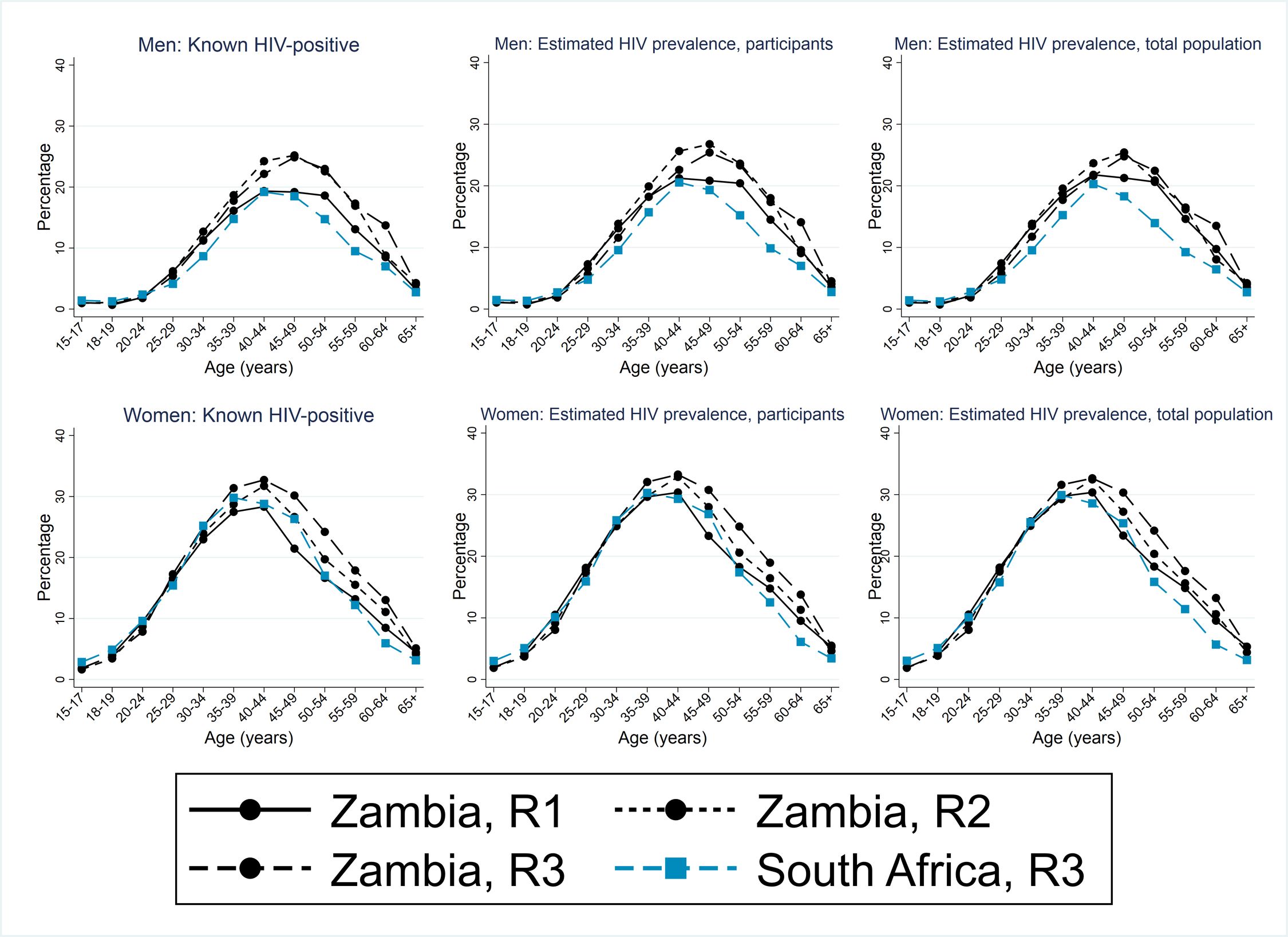

Supplement: S3 Fig — (TIF) [file pmed.1003067.s004.tif]

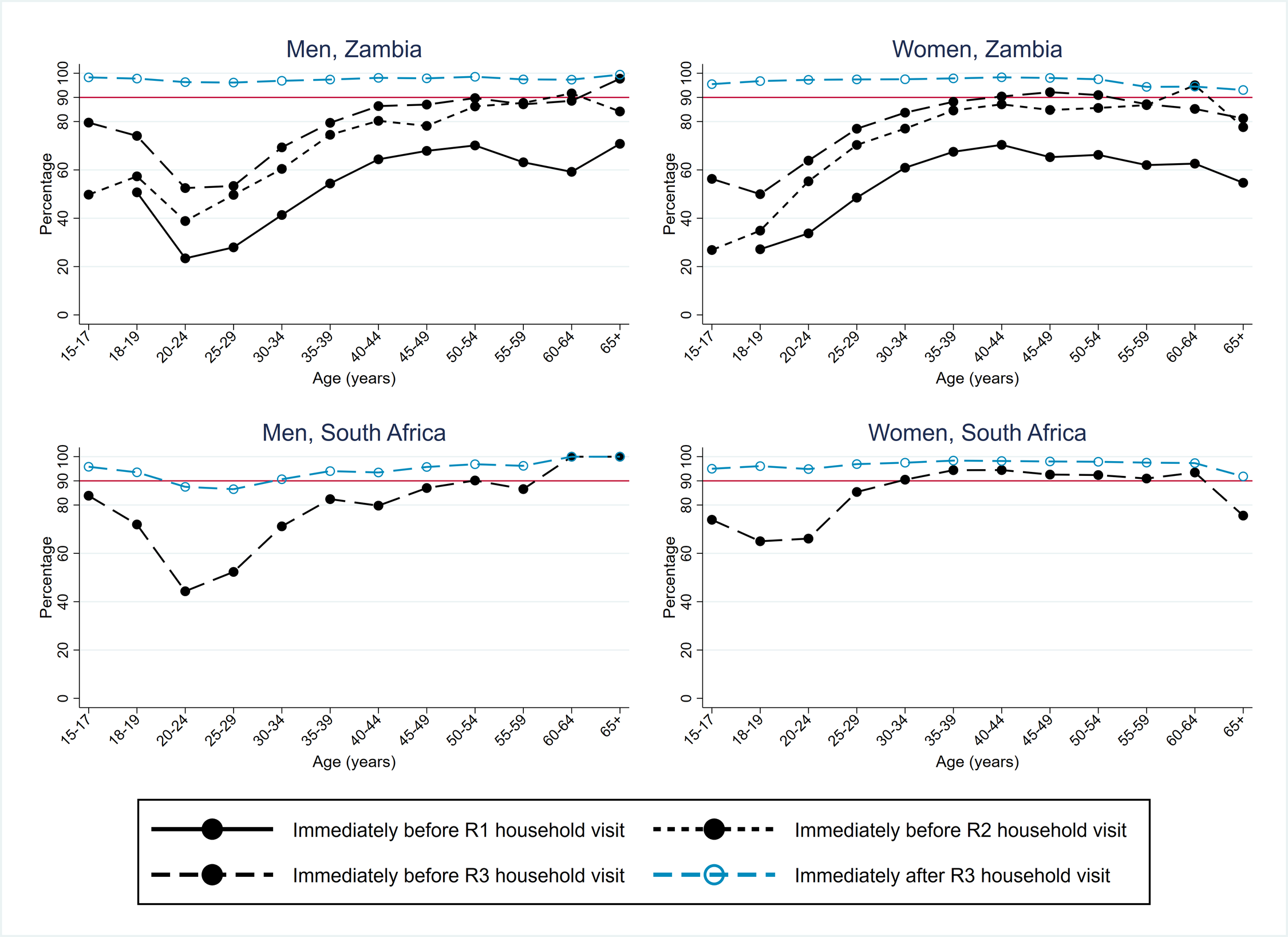

Supplement: S4 Fig — (TIF) [file pmed.1003067.s005.tif]

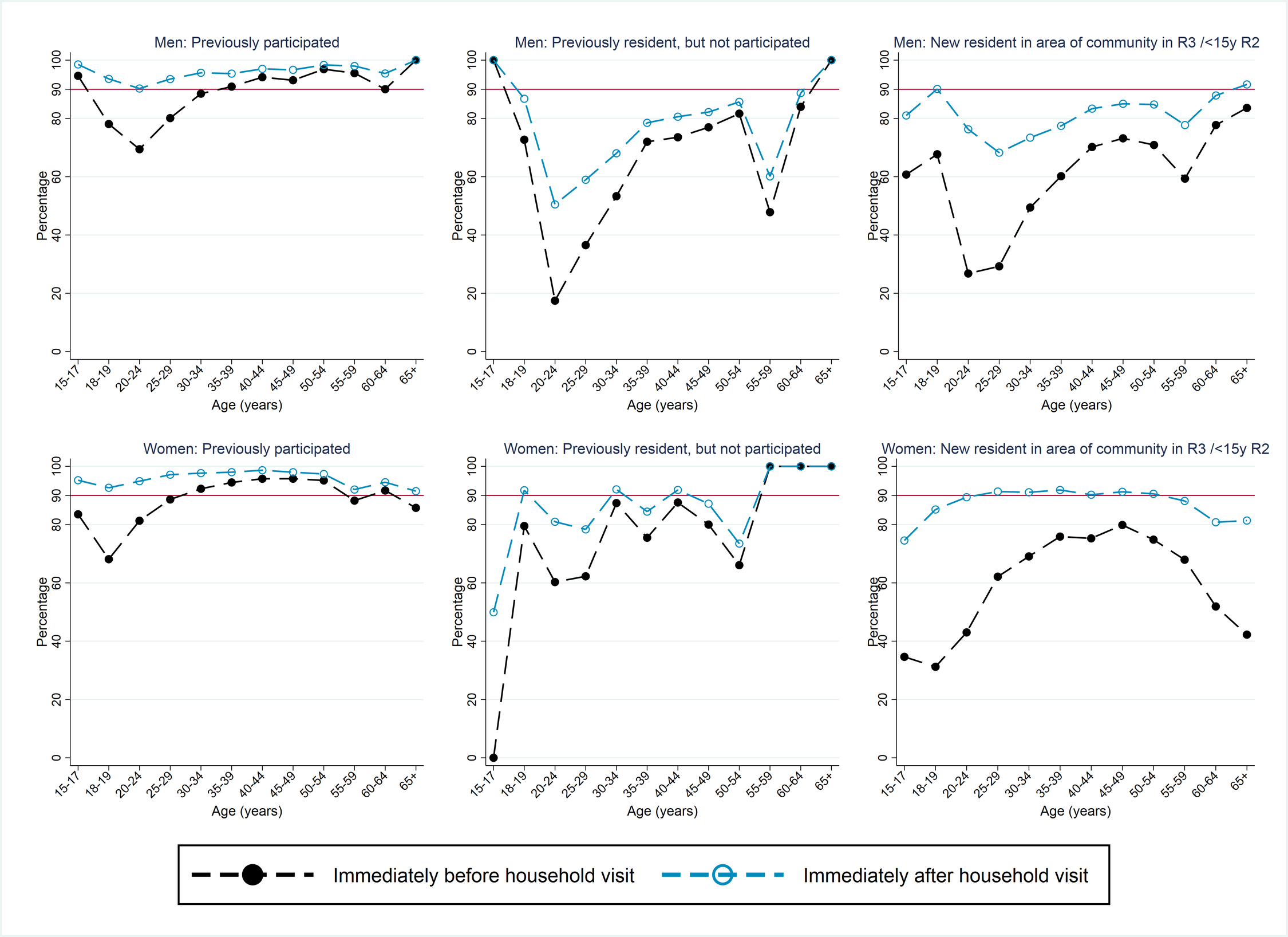

Supplement: S5 Fig — (TIF) [file pmed.1003067.s006.tif]

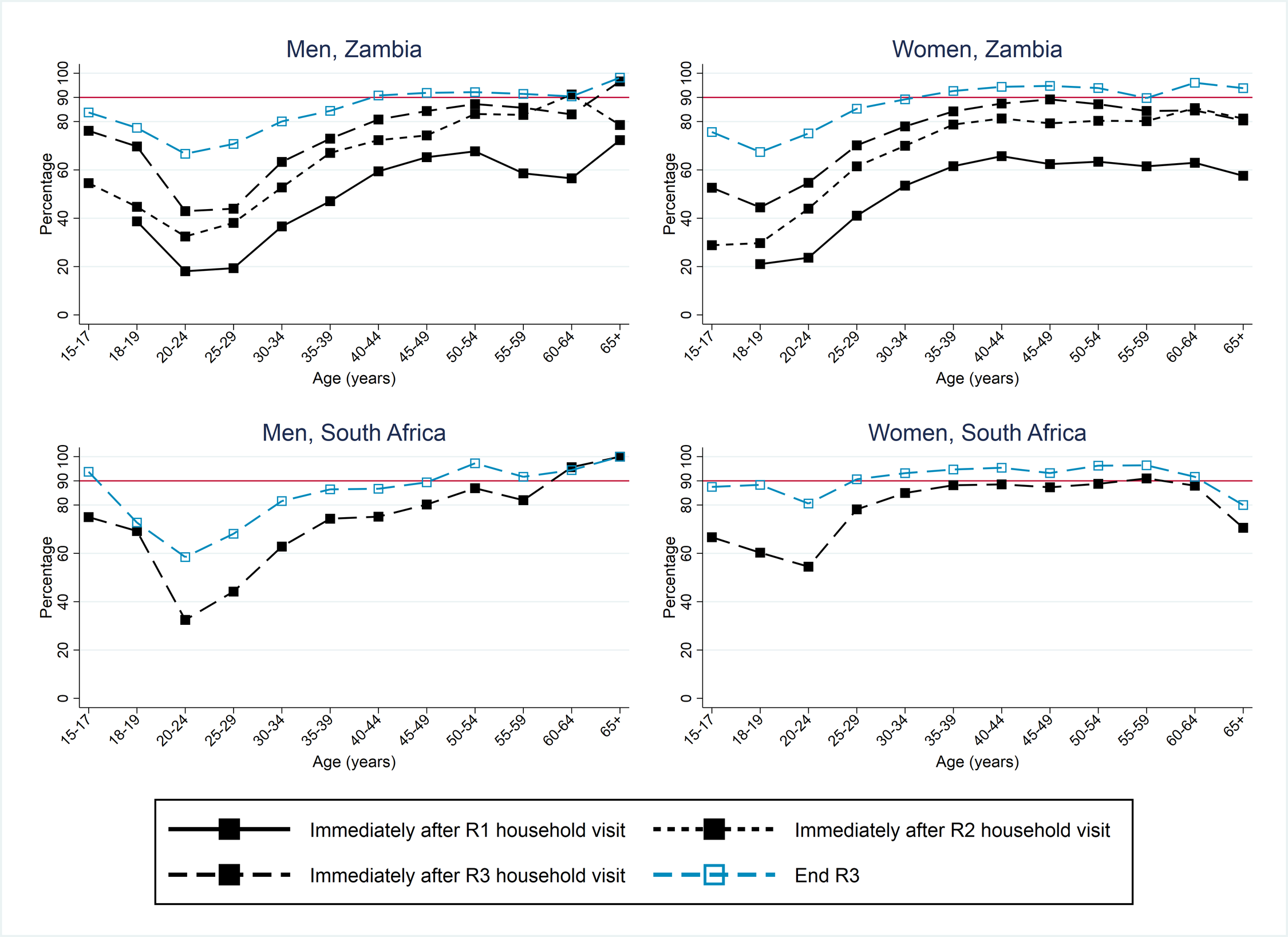

Supplement: S6 Fig — (TIF) [file pmed.1003067.s007.tif]

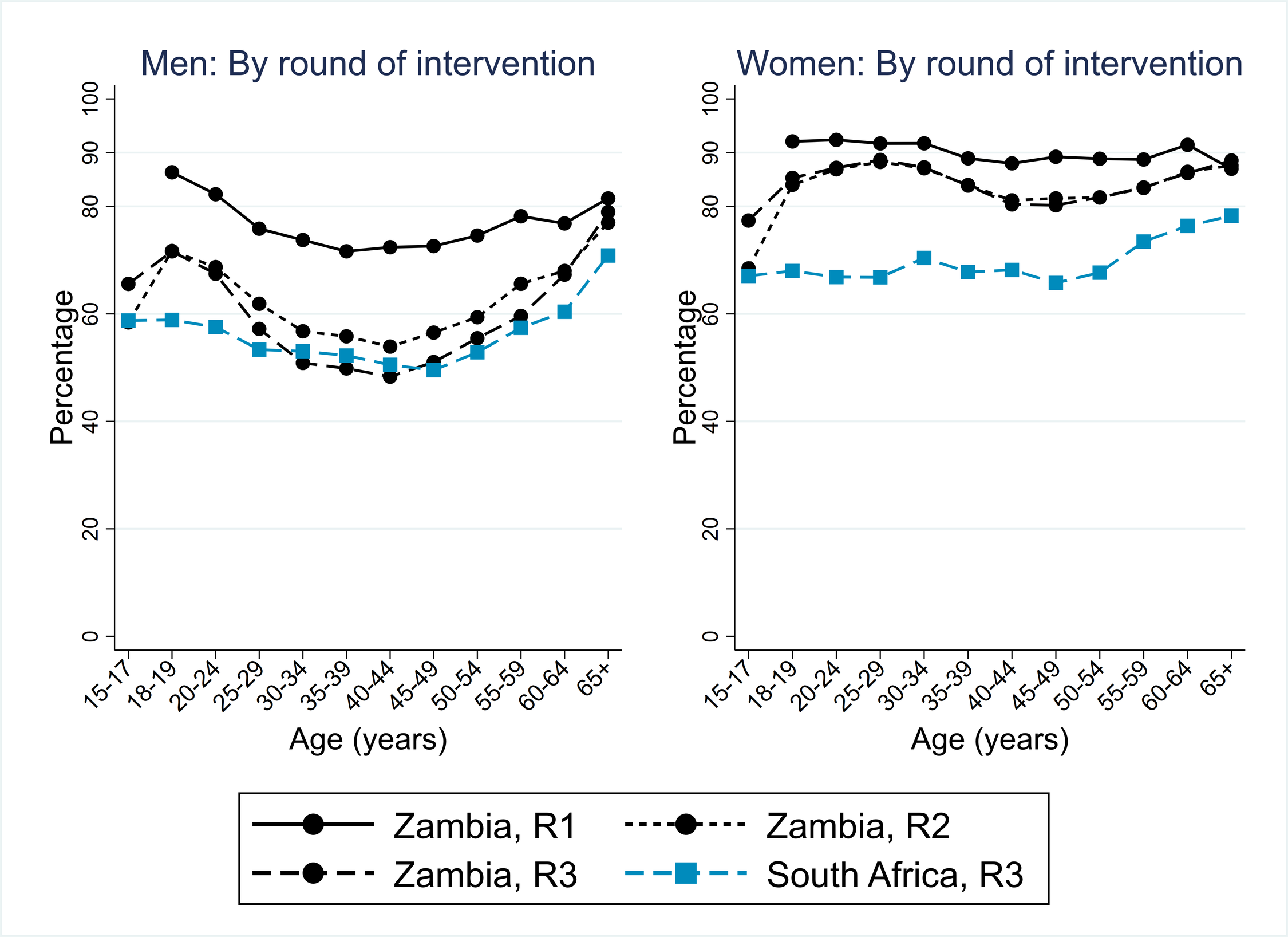

Supplement: S7 Fig — (TIF) [file pmed.1003067.s008.tif]

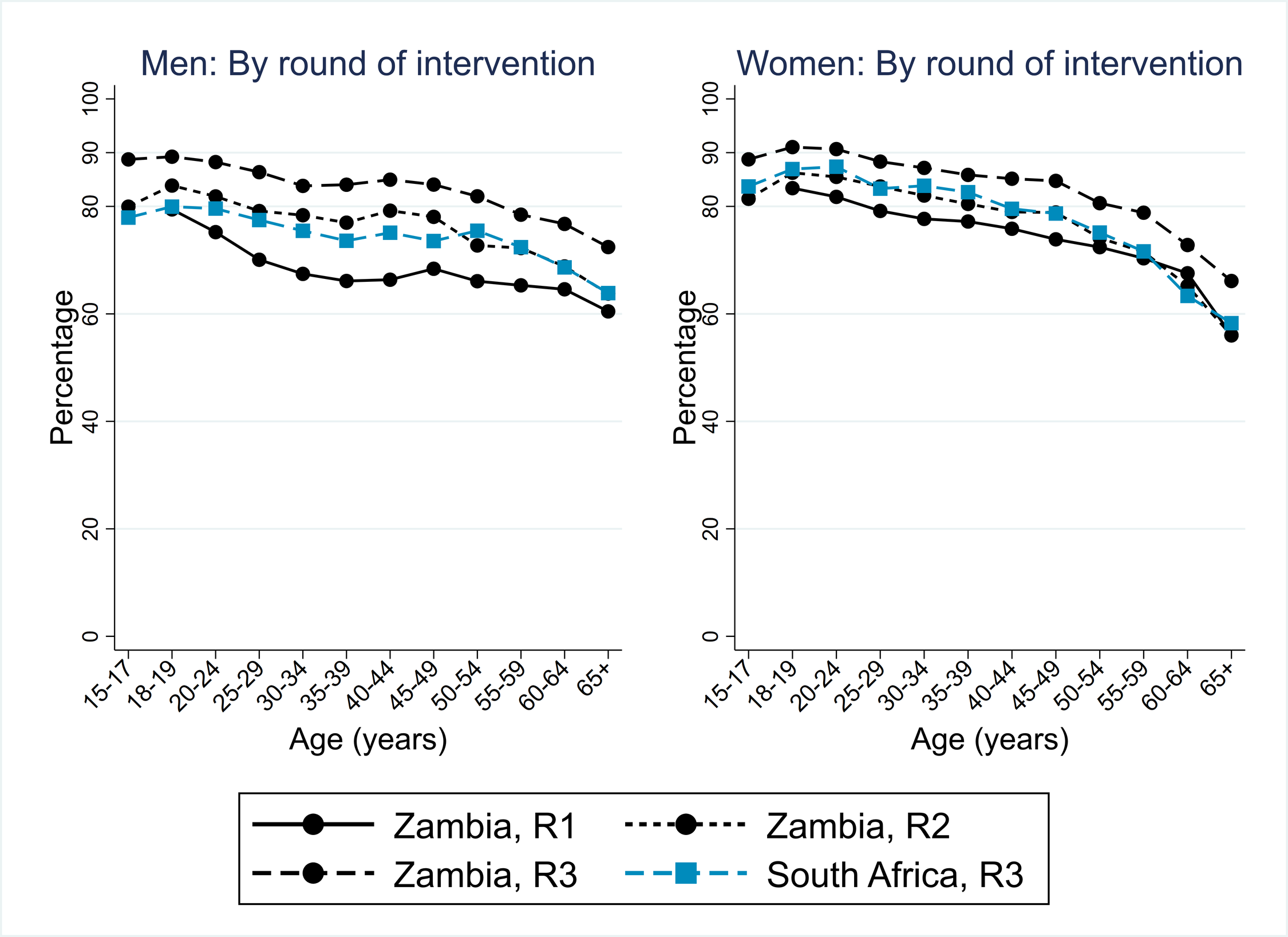

Supplement: S8 Fig — (TIF) [file pmed.1003067.s009.tif]

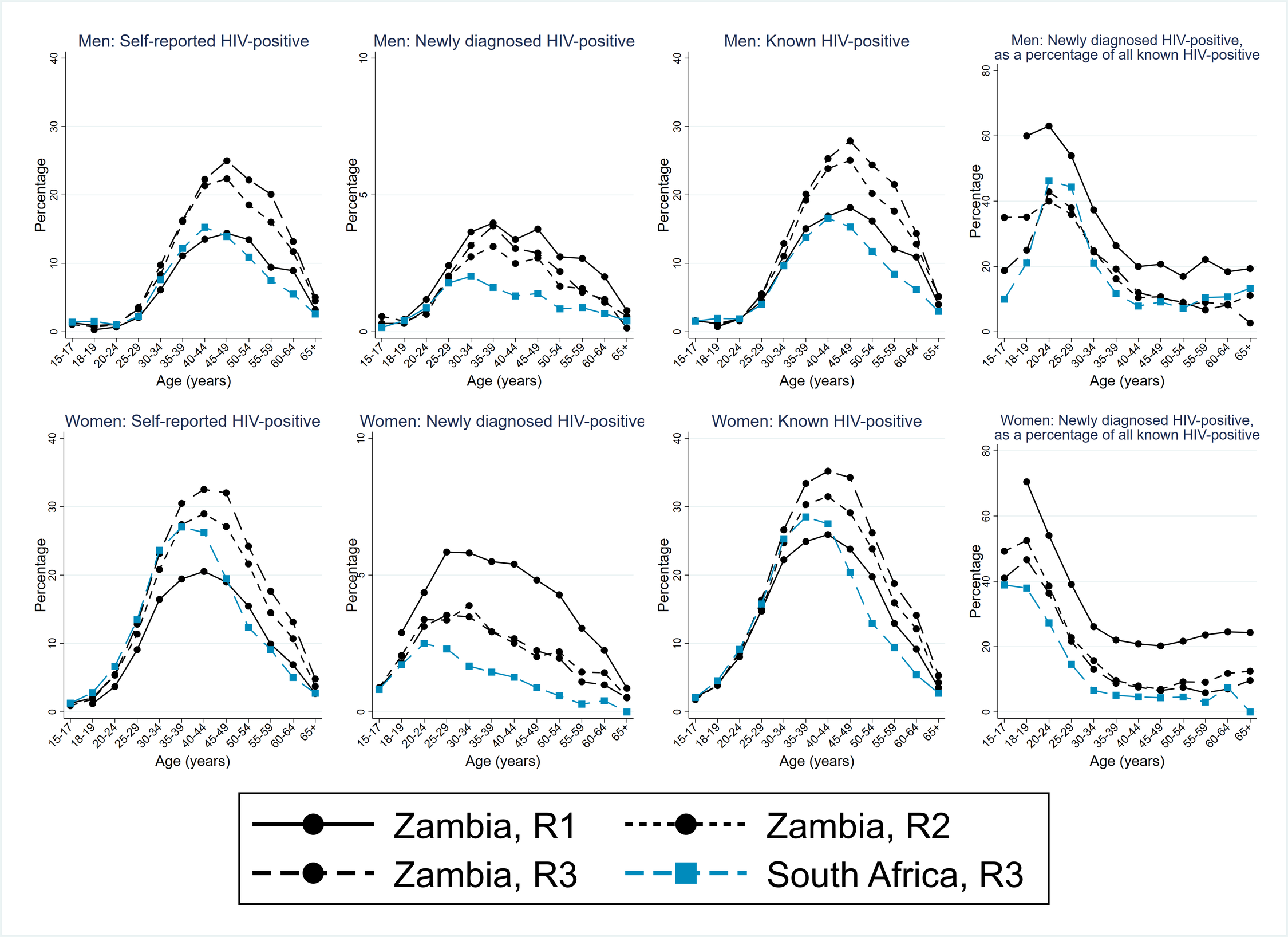

Supplement: S9 Fig — Zambian communities R1–R3 and SA communities R3, by sex and age group. (TIF) [file pmed.1003067.s010.tif]

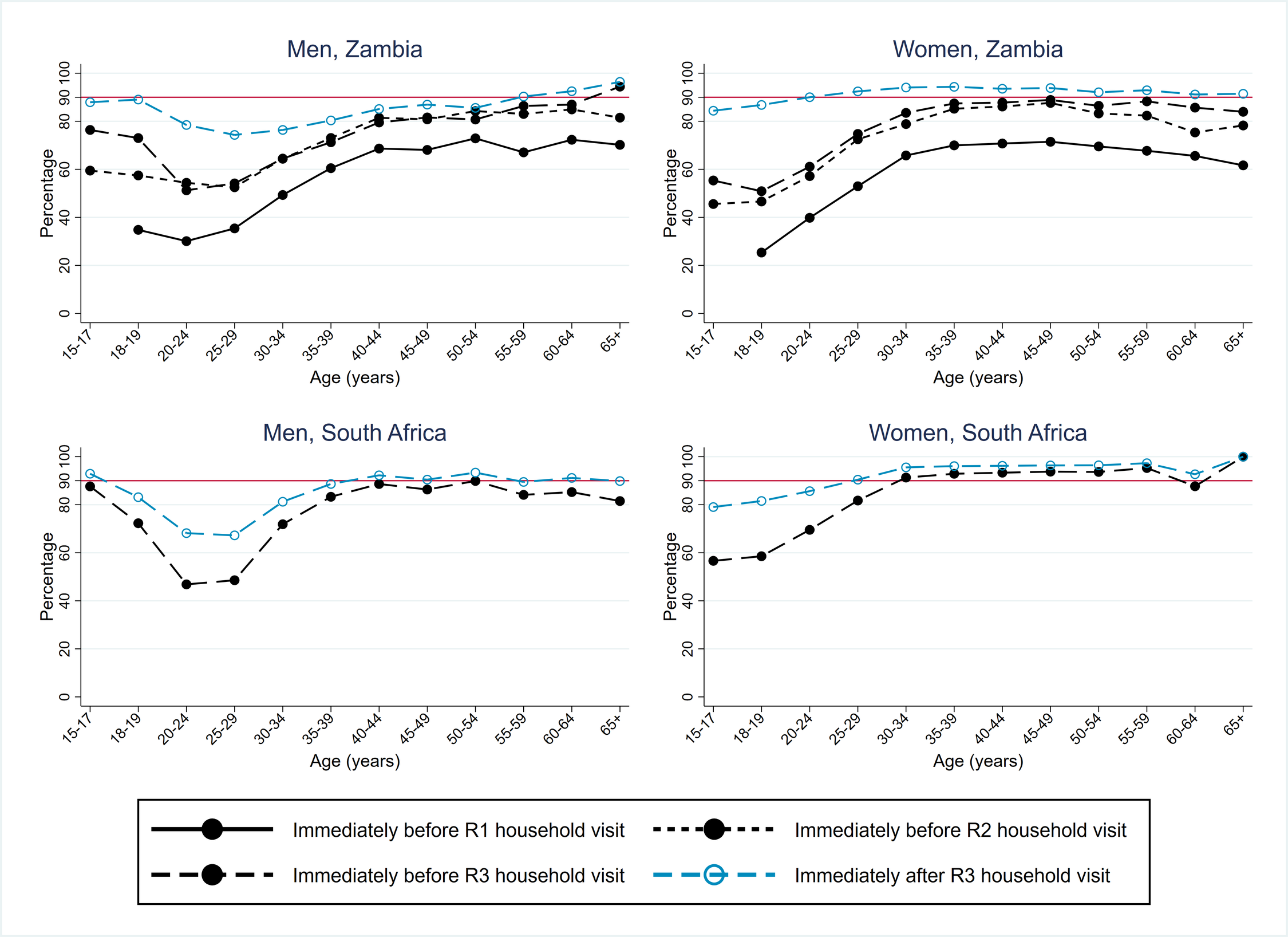

Supplement: S10 Fig — (TIF) [file pmed.1003067.s011.tif]

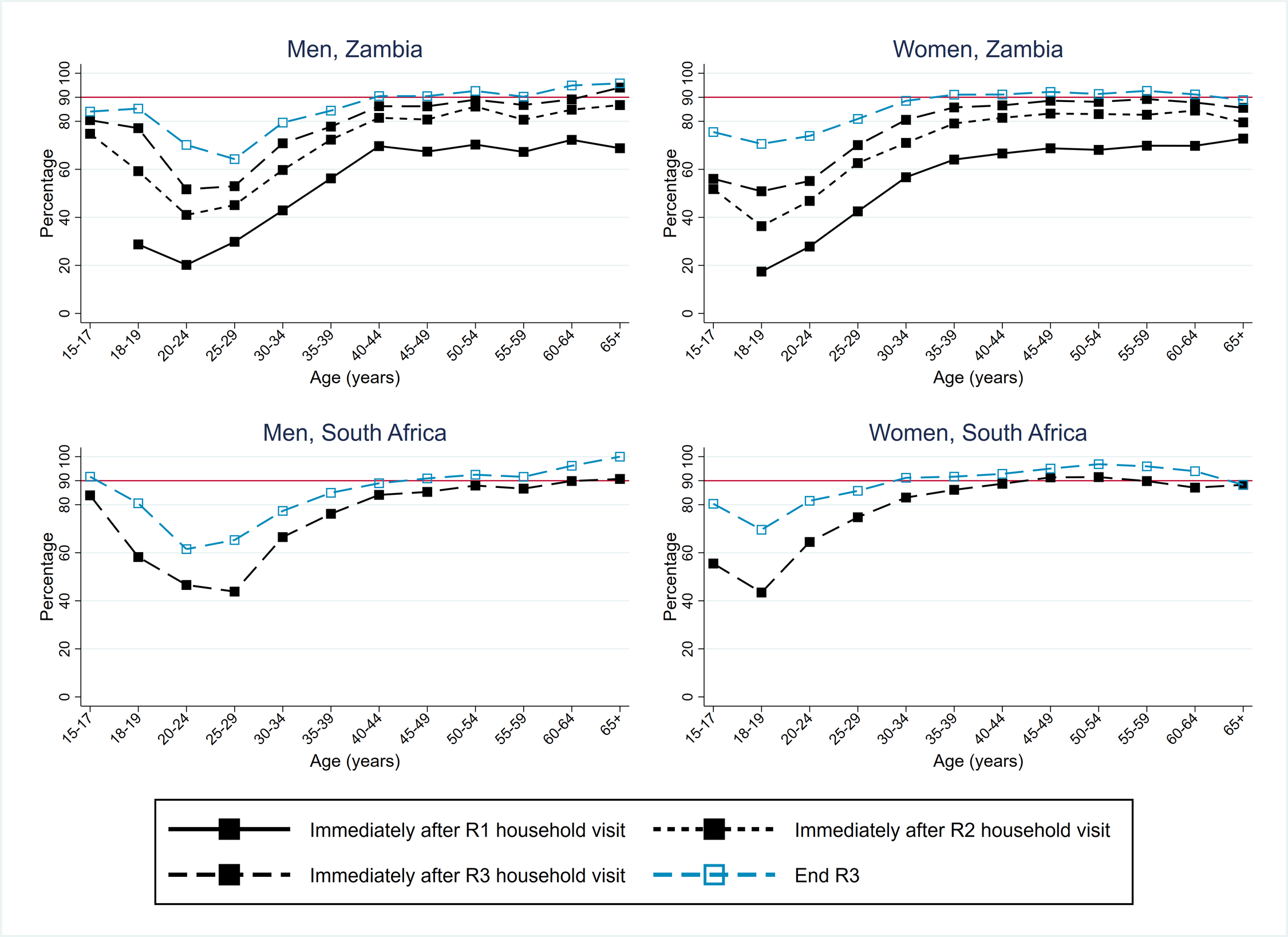

Supplement: S11 Fig — (TIF) [file pmed.1003067.s012.tif]

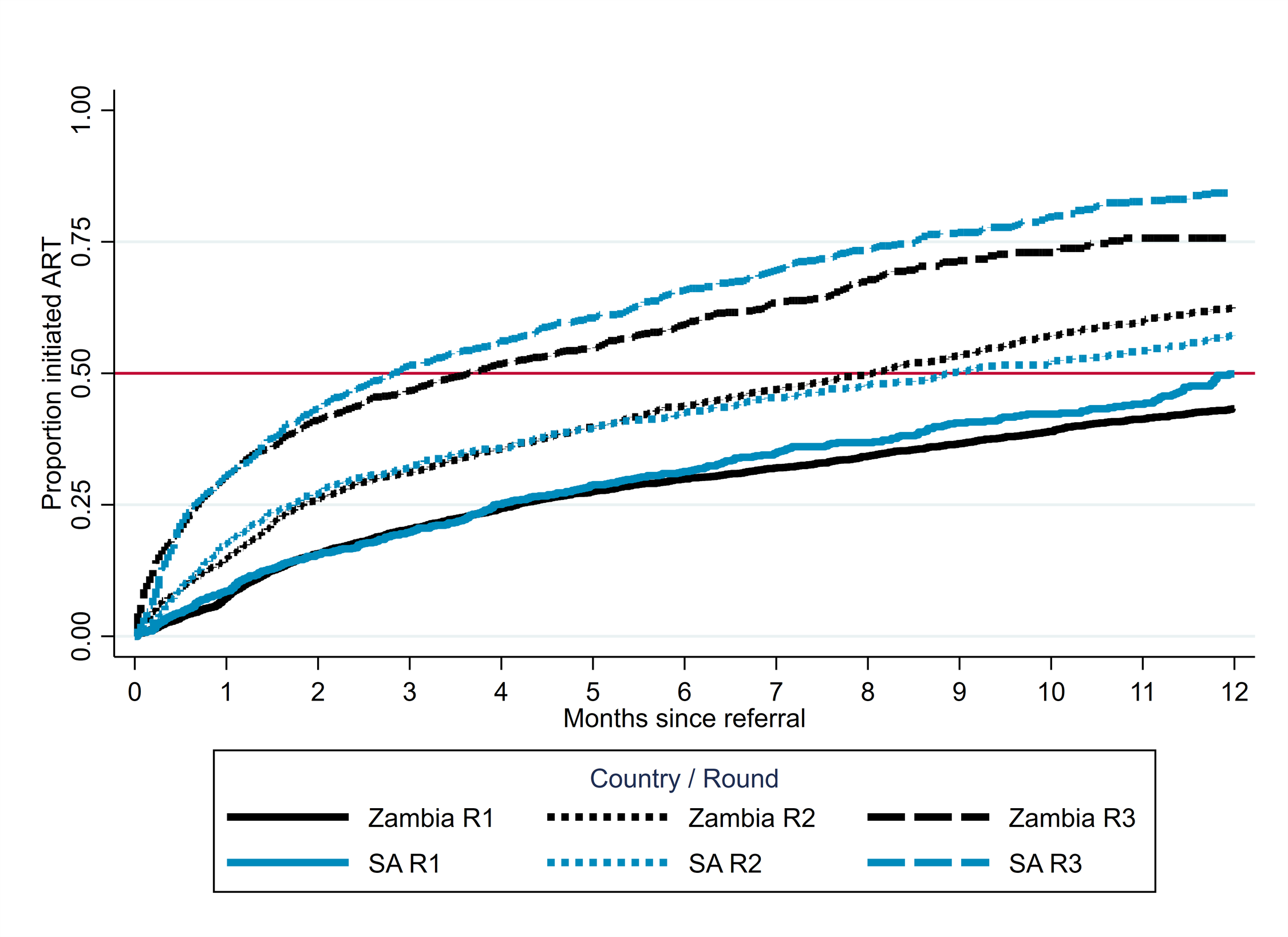

Supplement: S12 Fig — (TIF) [file pmed.1003067.s013.tif]
