# Supplementary material for: HIV testing and treatment coverage achieved after 4 years across 14 urban and peri-urban communities in Zambia and South Africa: An analysis of findings from the HPTN 071 (PopART) trial
Source: PLoS Med. 2020 Apr 2;17(4):e1003067. doi: 10.1371/journal.pmed.1003067 (PMC7117659; doi:10.1371/journal.pmed.1003067)
Supplement: S1 Table — Comparison of Arm A with Arm B communities, across 7 triplets of communities. (DOCX) [file pmed.1003067.s014.docx]

**S1 Table. Estimates of the percentage of HIV-positive individuals who knew their HIV-positive status immediately after the CHiP household visit of Round 3, among the estimated total population of HIV-positive individuals aged ≥15 years who were resident at the time of the CHiP household visit in Round 3 (first 90). Comparison of Arm A with Arm B communities, across 7 triplets of communities.**

|  |  | **Men** | | | | **Women** | | | |
| --- | --- | --- | --- | --- | --- | --- | --- | --- | --- |
|  |  | **Arm A** | | **Arm B** | | **Arm A** | | **Arm B** | |
| **Country** | **Triplet** | **%** | **n / N** | **%** | **n / N** | **%** | **n / N** | **%** | **n / N** |
| **Zambia** | **1** | **79.0** | 408 / 517 | **84.1** | 945 / 1124 | **91.4** | 952 / 1042 | **92.4** | 2190 / 2371 |
|  | **2** | **84.6** | 1164 / 1376 | **94.0** | 1106 / 1176 | **94.4** | 2186 / 2315 | **97.5** | 2048 / 2100 |
|  | **3** | **88.1** | 2759 / 3134 | **77.4** | 3010 / 3888 | **94.5** | 5209 / 5513 | **91.2** | 6107 / 6697 |
|  | **4** | **90.5** | 1102 / 1217 | **86.6** | 955 / 1102 | **95.2** | 2426 / 2547 | **94.2** | 1960 / 2081 |
| **SA** | **5** | **88.5** | 570 / 644 | **87.9** | 1263 / 1438 | **95.4** | 1350 / 1415 | **94.8** | 3294 / 3476 |
|  | **6** | **84.7** | 1716 / 2027 | **85.5** | 709 / 829 | **94.6** | 3956 / 4183 | **92.8** | 2051 / 2210 |
|  | **7** | **85.2** | 355 / 417 | **81.0** | 424 / 524 | **92.9** | 785 / 844 | **92.5** | 807 / 872 |
| **Zambia and SA** | **1-7** | **85.8** | 8074 / 9332 | **85.2** | 8413 / 10082 | **94.1** | 16865 / 17861 | **93.6** | 18457 / 19807 |
|  |  |  | | | | | | | |
|  |  | **Geometric mean** | **95% CI** | **Geometric mean** | **95% CI** | **Geometric mean** | **95% CI** | **Geometric mean** | **95% CI** |
| **Zambia** | **1-4** | **85.4** | 80.9 - 90.2 | **85.3** | 79.1 - 92.0 | **93.9** | 92.1 - 95.6 | **93.8** | 91.3 - 96.3 |
| **SA** | **5-7** | **86.1** | 80.8 - 91.7 | **84.8** | 77.7 - 92.5 | **94.3** | 92.3 - 96.3 | **93.4** | 90.5 - 96.3 |
| **Zambia and SA** | **1-7** | **85.7** | 82.3 - 89.3 | **85.1** | 80.4 - 90.1 | **94.0** | 92.7 - 95.4 | **93.6** | 91.7 - 95.5 |
|  |  | **Prevalence ratio**  **(A vs B)** | **95% CI** | **p-value** |  | **Prevalence ratio**  **(A vs B)** | **95% CI** | **p-value** |  |
|  |  | **1.01** | **0.94 - 1.08** | **0.81** |  | **1.01** | **0.98 - 1.03** | **0.58** |  |
