# Supplementary material for: HIV testing and treatment coverage achieved after 4 years across 14 urban and peri-urban communities in Zambia and South Africa: An analysis of findings from the HPTN 071 (PopART) trial
Source: PLoS Med. 2020 Apr 2;17(4):e1003067. doi: 10.1371/journal.pmed.1003067 (PMC7117659; doi:10.1371/journal.pmed.1003067)
Supplement: S2 Table — Comparison of Arm A with Arm B communities, across 7 triplets of communities. (DOCX) [file pmed.1003067.s015.docx]

**S2 Table. Estimates of the percentage of HIV-positive individuals who were on ART by the end of Round 3, among the estimated total population of HIV-positive individuals aged ≥15 years who knew their HIV-positive status immediately after the CHIP household visit of Round 3 and remained resident in the same CHiP zone at the end of Round 3 (second 90). Comparison of Arm A with Arm B communities, across 7 triplets of communities.**

|  |  | **Men** | | | | **Women** | | | |
| --- | --- | --- | --- | --- | --- | --- | --- | --- | --- |
|  |  | **Arm A** | | **Arm B** | | **Arm A** | | **Arm B** | |
| **Country** | **Triplet** | **%** | **n / N** | **%** | **n / N** | **%** | **n / N** | **%** | **n / N** |
| **Zambia** | **1** | **89.5** | 335 / 374 | **84.4** | 691 / 819 | **88.6** | 746 / 842 | **86.3** | 1616 / 1873 |
|  | **2** | **85.7** | 869 / 1014 | **90.5** | 874 / 966 | **86.6** | 1639 / 1893 | **92.0** | 1661 / 1806 |
|  | **3** | **88.5** | 2134 / 2411 | **84.4** | 2238 / 2652 | **90.7** | 3972 / 4382 | **84.8** | 4485 / 5290 |
|  | **4** | **86.7** | 875 / 1010 | **85.4** | 707 / 828 | **90.0** | 1983 / 2202 | **90.5** | 1512 / 1670 |
| **SA** | **5** | **88.4** | 473 / 535 | **87.8** | 1030 / 1172 | **93.5** | 1204 / 1287 | **91.6** | 2860 / 3121 |
|  | **6** | **84.5** | 1331 / 1575 | **82.3** | 533 / 647 | **91.9** | 3410 / 3710 | **88.4** | 1665 / 1884 |
|  | **7** | **80.0** | 270 / 338 | **79.8** | 306 / 383 | **88.1** | 645 / 732 | **91.2** | 682 / 749 |
| **Zambia and SA** | **1-7** | **86.2** | 6286 / 7257 | **84.9** | 6378 / 7468 | **89.9** | 13600 / 15049 | **89.2** | 14481 / 16392 |
|  |  |  | | | | | | | |
|  |  | **Geometric mean** | **95% CI** | **Geometric mean** | **95% CI** | **Geometric mean** | **95% CI** | **Geometric mean** | **95% CI** |
| **Zambia** | **1-4** | **87.6** | 83.6 - 91.8 | **86.1** | 81.9 - 90.6 | **89.0** | 86.2 - 91.9 | **88.3** | 85.0 - 91.9 |
| **SA** | **5-7** | **84.2** | 79.8 - 88.9 | **83.3** | 78.6 - 88.2 | **91.1** | 87.8 - 94.6 | **90.4** | 86.4 - 94.5 |
| **Zambia and SA** | **1-7** | **86.1** | 83.1 - 89.2 | **84.9** | 81.7 - 88.2 | **89.9** | 87.8 - 92.1 | **89.2** | 86.6 - 91.9 |
|  |  | **Prevalence ratio**  **(A vs B)** | **95% CI** | **p-value** |  | **Prevalence ratio**  **(A vs B)** | **95% CI** | **p-value** |  |
|  |  | **1.01** | **0.98 - 1.05** | **0.34** |  | **1.01** | **0.97 - 1.05** | **0.66** |  |
