# Supplementary material for: HIV testing and treatment coverage achieved after 4 years across 14 urban and peri-urban communities in Zambia and South Africa: An analysis of findings from the HPTN 071 (PopART) trial
Source: PLoS Med. 2020 Apr 2;17(4):e1003067. doi: 10.1371/journal.pmed.1003067 (PMC7117659; doi:10.1371/journal.pmed.1003067)
Supplement: S3 Table — Comparison of Arm A with Arm B communities, across 7 triplets of communities. (DOCX) [file pmed.1003067.s016.docx]

**S3 Table. Estimates of the percentage of HIV-positive individuals who were on ART by the end of Round 3, among the estimated total population of HIV-positive individuals aged ≥15 years who were resident at the time of the CHiP household visit in Round 3 and remained resident in the same CHiP zone at the end of Round 3 (ART coverage). Comparison of Arm A with Arm B communities, across 7 triplets of communities.**

|  |  | **Men** | | | | **Women** | | | |
| --- | --- | --- | --- | --- | --- | --- | --- | --- | --- |
|  |  | **Arm A** | | **Arm B** | | **Arm A** | | **Arm B** | |
| **Country** | **Triplet** | **%** | **n / N** | **%** | **n / N** | **%** | **n / N** | **%** | **n / N** |
| **Zambia** | **1** | **70.7** | 335 / 473 | **71.0** | 691 / 974 | **81.0** | 746 / 921 | **79.7** | 1616 / 2028 |
|  | **2** | **72.5** | 869 / 1199 | **85.1** | 874 / 1027 | **81.8** | 1639 / 2004 | **89.7** | 1661 / 1852 |
|  | **3** | **77.9** | 2134 / 2738 | **65.3** | 2238 / 3426 | **85.7** | 3972 / 4638 | **77.3** | 4485 / 5801 |
|  | **4** | **78.5** | 875 / 1116 | **73.9** | 707 / 957 | **85.8** | 1983 / 2312 | **85.3** | 1512 / 1774 |
| **SA** | **5** | **78.2** | 473 / 604 | **77.2** | 1030 / 1334 | **89.2** | 1204 / 1350 | **86.8** | 2860 / 3293 |
|  | **6** | **71.5** | 1331 / 1861 | **70.4** | 533 / 756 | **86.9** | 3410 / 3922 | **82.0** | 1665 / 2029 |
|  | **7** | **68.1** | 270 / 397 | **64.7** | 306 / 473 | **81.9** | 645 / 788 | **84.4** | 682 / 809 |
| **Zambia and SA** | **1-7** | **73.9** | 6286 / 8388 | **72.5** | 6378 / 8948 | **84.6** | 13600 / 15936 | **83.6** | 14481 / 17586 |
|  |  |  | | | | | | | |
|  |  | **Geometric mean** | **95% CI** | **Geometric mean** | **95% CI** | **Geometric mean** | **95% CI** | **Geometric mean** | **95% CI** |
| **Zambia** | **1-4** | **74.8** | 69.7 - 80.3 | **73.5** | 65.4 - 82.6 | **83.5** | 79.9 - 87.3 | **82.8** | 77.8 - 88.2 |
| **SA** | **5-7** | **72.5** | 66.9 - 78.6 | **70.6** | 61.6 - 80.8 | **85.9** | 81.6 - 90.5 | **84.4** | 78.5 - 90.7 |
| **Zambia and SA** | **1-7** | **73.8** | 70.0 - 77.8 | **72.2** | 66.1 - 78.9 | **84.5** | 81.7 - 87.4 | **83.5** | 79.7 - 87.5 |
|  |  | **Prevalence ratio**  **(A vs B)** | **95% CI** | **p-value** |  | **Prevalence ratio**  **(A vs B)** | **95% CI** | **p-value** |  |
|  |  | **1.02** | **0.93 - 1.12** | **0.59** |  | **1.01** | **0.96 - 1.07** | **0.61** |  |
