# Supplementary material for: HIV testing and treatment coverage achieved after 4 years across 14 urban and peri-urban communities in Zambia and South Africa: An analysis of findings from the HPTN 071 (PopART) trial
Source: PLoS Med. 2020 Apr 2;17(4):e1003067. doi: 10.1371/journal.pmed.1003067 (PMC7117659; doi:10.1371/journal.pmed.1003067)
Supplement: S2 Data — (DOCX) [file pmed.1003067.s018.docx]

**S2 Data: Definition of variables on aggregated dataset**

| Variable name | Variable description | Coding |
| --- | --- | --- |
| country | Country of residence | 1 = Zambia; 2 = South Africa |
| round | Intervention round | 1,2,3. For South Africa, round 3 only |
| arm | Trial arm | 1=Arm A; 2=Arm B |
| triplet | Study triplet | 1-7 |
| community | Community of residence | 1-14 |
| gender | Gender | 1= Male; 2=Female |
| agegroup | Age group (years) | 0=15-17; 1=18-19; 2=20-24; 3=25-29; 4=30-34; 5=35-39; 6=40-44; 7=45-49; 8=50-54; 9=55-59; 10=60-64; 11=65+ |
| residence | Participation and residency in previous rounds, for Rounds 2 and 3 only; coded for Zambian communities only | Zambia, Round 1 (R1) and South Africa Round 3 (R3)  99 = code for all individuals, in absence of prior information  Zambia, Round 2 (R2):  0=Resident in same CHiP zone in R1, did not participate in R1;  1=Participated in R1, self-reported HIV-positive;  2=Participated in R1, tested HIV-positive;  3=Participated in R1, tested HIV-negative;  4=Participated in R1, but did not self-report HIV-positive and did not accept the offer of HIV testing;  5=Newly resident in the CHiP zone in which they were resident at the start of R2, and/or aged <18 years at time of R1  Zambia, Round 3 (R3):  0=Resident in same CHiP zone in R1 & R2, did not participate in either round;  1=Participated in R1 &/or R2, self-reported HIV-positive in R1 &/or R2;  2=Participated in R2, tested HIV-positive in R2;  3=Participated in R1 &/or R2, tested HIV-negative in R1 &/or R2, did not self-report or test HIV-positive in R1 or R2;  4=Participated in R1 &/or R2, did not self-report HIV-positive and did not accept the offer of HIV testing in either round;  5=Newly resident in the CHiP zone in which they were resident at the start of R3, and/or aged <15 years at time of R2;  9=Resident in the same CHiP zone in R2 (as the zone in which they were resident in R3) but did not participate in R2, not resident in this zone in R1 |
| prop_hh_enumerated | Proportion of households that consented to enumeration, among visited households | Estimated for each combination of round and community |
| enumerated | Enumerated as a household member | Count of individuals |
| participated | Participated in intervention (Consented to participate, and had health counselling done) | Count of individuals |
| self_report_hivpos | Self-reported HIV-positive | Count of individuals |
| nd_hivpos | Newly diagnosed HIV-positive = tested HIV-positive among individuals who participated | Count of individuals |
| know_hivstatus | Knows HIV status immediately after current round’s annual visit (defined as self-reported HIV-positive, tested with CHiPs in current round, or as part of the annual visit they reported an HIV-negative test result in the previous 3 months) | Count of individuals |
| known_hivpos | Known HIV-positive (self-reported HIV-positive plus tested HIV-positive) following the annual round visit, among individuals who participated | Count of individuals |
| tested_for_hiv | Tested for HIV, among those who had health counselling done and also among those who self-tested (Zambia, R3 only) but did not have health counselling done | Count of individuals |
| tested_hivpos | Tested HIV-positive, among those who had health counselling done and also among those who self-tested (Zambia, R3 only) but did not have health counselling done | Count of individuals |
| on_art_baseline | Self-reported on ART at time of annual visit | Count of individuals |
| known_hivpos_resident_end_round | Resident in the same area (CHiP zone) of the community at the end of the round, according to last information collected in the round, among individuals who participated in the current round and were known to be HIV-positive | Count of individuals |
| on_art_end_round | On ART at the end of the round, among those who participated in the current round and were known to be HIV-positive, and were still resident in the same area of the community at the end of the round according to the last information collected in the round | Count of individuals |
